# Precise mode control of laser-written waveguides for broadband, low-dispersion 3D integrated optics

**DOI:** 10.1038/s41377-024-01473-7

**Published:** 2024-06-04

**Authors:** Yuying Wang, Lijing Zhong, Kuen Yao Lau, Xuhu Han, Yi Yang, Jiacheng Hu, Sergei Firstov, Zhi Chen, Zhijun Ma, Limin Tong, Kin Seng Chiang, Dezhi Tan, Jianrong Qiu

**Affiliations:** 1 College of Optical Science and Engineering, Zhejiang University, 310027 Hangzhou, China; 2 Institute of Light+X Science and Technology, College of Information Science and Engineering, Ningbo University, 315211 Ningbo, China; 3 School of Optoelectronic Science and Engineering & Collaborative Innovation Center of Suzhou Nano Science and Technology, Soochow University, 215006 Suzhou, China; 4 Prokhorov General Physics Institute of the Russian Academy of Sciences, Dianov Fiber Optics Research Center, 38 Vavilov str., Moscow, 119333 Russia; 5 Zhejiang Lab, 311121 Hangzhou, China; 6 College of Materials Science and Engineering, Key Laboratory of Advanced Materials of Yunnan Province, Kunming University of Science and Technology, 650093 Kunming, Yunnan China; 7 Department of Electrical Engineering, City University of Hong Kong, 83 Tat Chee Avenue, Kowloon, Hong Kong SAR, China; 8 School of Materials Science and Engineering, Zhejiang University, 310027 Hangzhou, China

**Keywords:** Laser material processing, Silicon photonics

## Abstract

Three-dimensional (3D) glass chips are promising waveguide platforms for building hybrid 3D photonic circuits due to their 3D topological capabilities, large transparent windows, and low coupling dispersion. At present, the key challenge in scaling down a benchtop optical system to a glass chip is the lack of precise methods for controlling the mode field and optical coupling of 3D waveguide circuits. Here, we propose an overlap-controlled multi-scan (OCMS) method based on laser-direct lithography that allows customizing the refractive index profile of 3D waveguides with high spatial precision in a variety of glasses. On the basis of this method, we achieve variable mode-field distribution, robust and broadband coupling, and thereby demonstrate dispersionless LP_21_-mode conversion of supercontinuum pulses with the largest deviation of <0.1 dB in coupling ratios on 210 nm broadband. This approach provides a route to achieve ultra-broadband and low-dispersion coupling in 3D photonic circuits, with overwhelming advantages over conventional planar waveguide-optic platforms for on-chip transmission and manipulation of ultrashort laser pulses and broadband supercontinuum.

## Abstract (web summary)

We delineate a femtosecond laser-direct writing method that allows customizing the refractive index profile (both shape and distribution) of 3D waveguides in a variety of glasses with high spatial precision.

## Graphical abstract

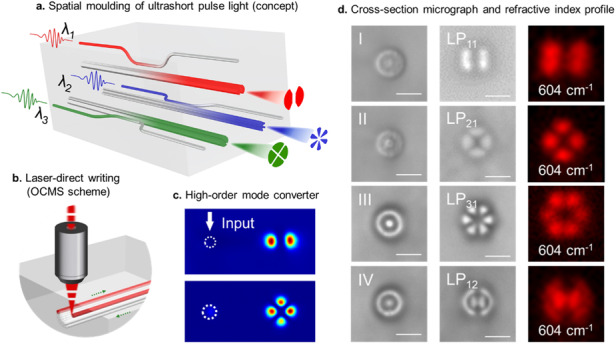

## Introduction

Waveguide optics incorporating controllable refractive index (RI) profile allow for a new physical degree of freedom to design and manipulate more complex light modes, including in fibers^1^ or integrated photonics chips^2^. However, state-of-the-art integrated optics are technically based on similar etch and deposition processes of 2D planar^3–5^, offering only size and few cross-sectional shapes (mainly rectangles and trapezoids) as available parameters for waveguide mode control, facing critical challenges in manipulating centrosymmetric modes, transforming beam polarization state, and high manufacturing complexity. In contrast, femtosecond laser-direct writing (FLDW) is a reliable technology to tailor waveguide cross-sectional shape^6,7^, allowing flexible transformation of the cross-section along the waveguide^8,9^. However, whether based on traditional planar lithography or the current FLDW technology, fine control over the RI distribution of waveguides still remains out of reach, primarily due to the absence of a universal technical mean that can space-selectively change the RI with high spatial resolution, resulting in a huge obstacle to precise control of mode field distribution and optical coupling especially in 3D waveguides.

**Fig. 1:**
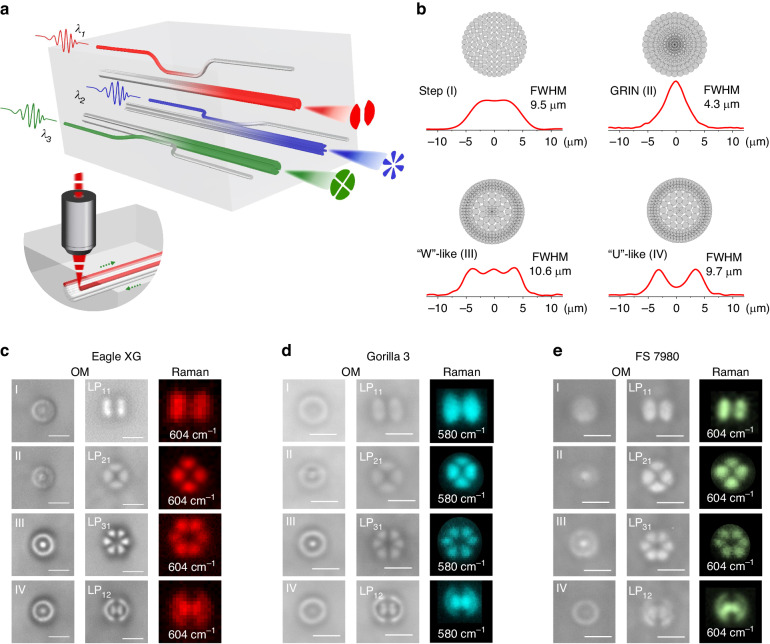
Spatial moulding of ultrashort pulse light based on OCMS waveguide with variable cross-section and RI profile. **a** Conceptual scheme for ultrashort pulses with different central wavelengths (λ_1_, λ_2_, λ_3_) to undergo spatial mode conversion into high-order mode pulses. Schematic illustration of a laser-written waveguide employing the OCMS approach (down inset). **b** Design diagram of scanline overlapping schemes (upper) for four types of fundamental-mode waveguides with measured index distributions (down) of step (I), GRIN (II), “W”-like (III) and “U”-like (IV), respectively. **c**–**e** Optical micrographs (OM) of four fundamental-mode waveguides (left), four higher-order mode waveguides for LP_11_, LP_21_, LP_31_, and LP_12_ modes (middle) in three typical glasses (Eagle XG, Gorilla 3 and fused silica FS 7980), as well as Raman mappings of four higher-order mode waveguides (right). Scale bars are 10 μm

In this work, we present a major step forward in solving this long-standing optical problem by developing a precise FLDW method, namely overlap-controlled multi-scan (OCMS) method, for controlling the RI profile of 3D waveguides by accurately taming the thermal accumulation effect within the laser irradiation region, and amazingly being applicable to various glasses regardless of their chemical composition. With OCMS, we present the first arbitrary RI fabrication process that achieves submicron spatial resolution and RI resolution on the order of 10^−5^. The versatility of OCMS technology enables geometries and RI configurations previously unachievable with traditional FLDW. Furthermore, building on this technological advancement, we propose a chip-scale pulsed light manipulation platform in glass which can realize arbitrary spatial mode manipulation of ultra-short laser pulses and ultra-broadband supercontinuum. This is achieved by precisely accessing and tailoring the spatial light distribution of an ultrashort pulse beam through the spatial mode transformation over an ultra-broad spectrum. Specifically, we propose to tailor the spatial distribution of the optical field by means of a highly customizable spatial-mode converter with exceptionally high spatial resolution, thus enabling conformal transmission of pulsed light in the time domain while performing mode manipulation in the spatial domain. As conceptually shown in Fig. 1a, we delineate a 3D glass chip that exceptionally enables adiabatic high-order mode conversion, conformal laser pulse transmission, ultra-broadband operation with centered wavelengths covering visible and near-infrared spectrum. Overall, this work offers a general approach that deliberately manipulates the spatial entities of the temporal optical field in an integrated 3D glass chip at will.

## Results

### OCMS scheme for tailoring waveguide RI profiles

Mode manipulation in the integrated photonics is inherently linked to the control of mode-field distribution and mode coupling along the light-guiding path, which is technically limited by the spatial positioning accuracy and the control precision of the waveguide RI profile. In FLDW waveguides, the core factor that limits the control accuracy of the shape and RI distribution of the laser modification area is the complicated thermal accumulation and diffusion^10,11^, and self-focusing effects^12,13^ under the high-repetition rate femtosecond laser irradiations. In this work, we explore a previously untapped processing window with pulse energies 1.0–1.5 times greater than the optical breakdown threshold but well below the self-focusing threshold, where the shape and size of the modified region is determined by the focal volume of the target (see Supplementary text S1 and Fig. S1 for more details). In other words, it is free of thermal diffusion and filamentation effects and thus the laser-induced modification is localized within the focal region, which is crucial for finely manufacturing waveguide coupling structures to achieve a high coupling ratio. Furthermore, in order to overcome its shortcomings of small size and low RI contrast, we propose to control the RI change value at different spatial spots by developing a general fabrication strategy that spatially selectively changes the overlap ratio (OR) of adjacent written scanlines, i.e. OCMS, as schematically illustrated in Fig. 1a (inset) and Fig. S2a. The underlying mechanism of OCMS changing the RI is based on a well-established fact in the multi-scan FLDW technique that the OR of adjacent scanlines are positively correlated with the obtained RI change^14^. In the writing process, by closely stacking scan-lines at specific OR distribution (Fig. 1a inset), the OCMS method creates waveguides with designed RI distribution (see Supplementary text S2). The spatial resolution when constructing waveguides using the OCMS method is determined by the size of the individual scanline. In this work, an oil-immersed objective with a high NA of 1.30 is used to minimalize the dimension of a single scanline. We exploited a special processing window with low pulse energy of 20–30 nJ, 1.0–1.5 times larger than the structural modification threshold but well below the self-focusing threshold^12^, which makes a tiny RI change structure with a size smaller than the diffraction limit of ~1.2 μm of the objective, and avoids focus position shifts due to self-phase modulation and self-focusing effects (see Supplementary text S1). The sub-diffraction limit-sized scanline gives higher resolution in control over cross-sectional shape and spatial positioning. As shown in Fig. 1b, a single scan-line with a diameter of 800 nm is used as the basic structural unit for constructing the waveguide cross-section, the spacing between adjacent scanline centers is as low as 50 nm with the help of high-precision motion control, which means that the smallest width of the RI modification unit can be controlled down to 50 nm nearly an order of magnitude lower than previously (>0.4 μm)^8,9,14–16^. Furthermore, the proposed mechanism to make precise RI control by fine engineering thermal accumulation effects can be generalized to various transparent materials, including commercial available screen glasses Eagle XG (Fig. 1c) and Gorilla 3 (Fig. 1d), fused silica glass FS-7980 (Fig. 1e). Remarkably, the OCMS method enables not only positive but also negative RI control, such as in the alkali-rich glass K9 (see Supplementary text S3 for details); or further, the OCMS approach allows for arbitrarily customizing the degree of laser-induced thermal modifications with exceptionally high resolution, providing an efficient route to produce submicron or nanoscale structural modifications in glasses beyond current thermal FLDW techniques^17–19^. Additionally, the proposed OCMS processing strategy is not limited to the objective lens used, but can be easily extended to any objective lens according to practical applications, by carefully tailoring the RI structure of a single scanline through laser beam shaping methods, including astigmatic beam shaping^20,21^, aberration correction^22^ and manually added spherical aberration^9^.

To demonstrate the RI control capability of the OCMS method, we design four circular waveguides with the same 10 µm diameter but different OR distributions as step, GRIN, W-like and U-like profiles (Fig. S2c). The obtained RI distributions are measured by the Raman mapping (see Supplementary text S4 for detailed analysis), and the results are highly consistent with designed OR profiles (Fig. 1b). The full width at half maximum (FWHM) of waveguide cross-sections are changed from 4.3 to 10.6 μm due to the remarkable difference in their RI distributions. A numerical method employing the Inverse-Helmholtz equation (see Supplementary text S5 for detailed analysis), reveals that the OCMS method enables a high accuracy of ~5.6 × 10^−5^ in the control of RI change. Moreover, the OCMS method can delineate cross-sectional shapes with higher spatial precision than before, which makes complex waveguide geometry mapping and arbitrary cross-sectional transformations possible. Figure 1c–f illustrates waveguides with centrosymmetric cross-sections for the purpose of selecting on-demand Laguerre–Gaussian modes, where their cross-sectional shapes and RI distributions are made to match the intensity distribution of LP_11_, LP_21_, LP_31_ and LP_12_ modes. According to the mode competition theory in laser optics^23,24^, these high-order mode waveguides act as intensity- or phase- modulating elements for selecting the corresponding mode while suppressing other unwanted modes in the light propagation path, thereby enabling mode conversion that can effectively convert the fundamental LP_01_ mode to the corresponding high-order mode, for example converts to LP_11_ mode with a mode extinction ratio (MER) of ~5 dB (see Supplementary text S6). Furthermore, as discussed below, their mode purity can be dramatically improved by designing corresponding mode-selective couplers. Therefore, based on the fine engineering of thermal accumulation in laser processing, the proposed OCMS scheme, for the first time among FLDW techniques allows accurate and precise control of the cross-sectional shape and RI distribution, thereby providing new degrees of freedom for manipulating the mode field and coupling of 3D waveguide circuits.

### Variable mode-field-diameter (MFD) of single-mode OCMS waveguide

Generally, the multi-scan scheme in FLDW techniques has strong processing robustness against external disturbances, which can offset the influence of laser power fluctuation and environmental vibrations on the uniformity of the waveguide, thereby reducing the generation of scattering centers^14,16,25^. Here, beyond this capability, the developed OCMS technique enables precise control over mode field distribution and mode circularity by providing great flexibility and robustness in the customization of both RI distribution and cross-section shape, thereby achieving excellent fiber-waveguide interconnection with low coupling loss approaching theoretical limits.

**Fig. 2:**
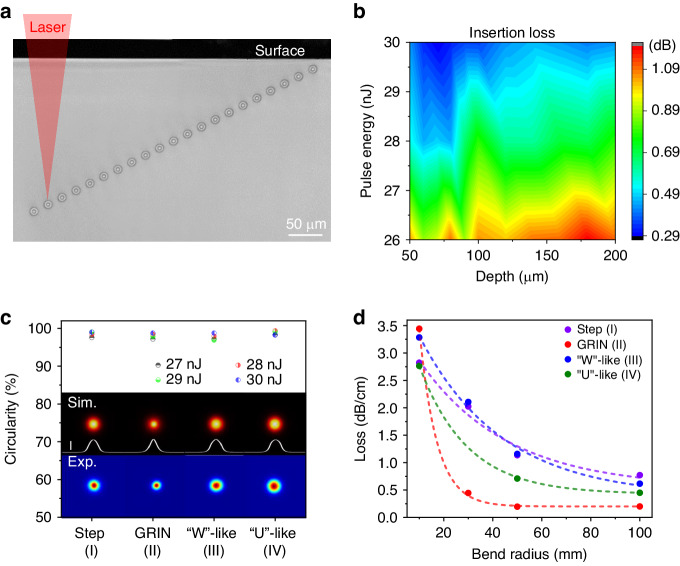
OCMS waveguides characterization. **a**, **b** Optical micrographs of multiple GRIN waveguides written to depths of 10–200 μm and the corresponding insertion losses at 1550 nm, where the applied pulse energy is varied from 26 to 30 μJ. **c**, **d** Mode circularity and bending loss of four types of single-mode waveguides written with 29 μJ pulse energy and 5 mm/s scan speed

To characterize the waveguide performance, we compare multiple GRIN waveguides written at different depths (Fig. 2). Under the same laser processing conditions of 29 μJ pulse energy and 5 mm/s scan speed, these GRIN waveguides exhibit excellent consistency in microscopic shape and brightness contrast over a wide range of writing depths from 10 to 200 μm (Fig. 2a), demonstrating strong writing depth robustness of the OCMS approach. In application, the GRIN distribution of these waveguides makes them highly compatible with commercial GRIN fibers by providing a record low insertion loss (*L*_*i*_) of 0.29 dB of a 10 mm long waveguide at 1550 nm (Fig. 2b). The waveguide insertion loss (*L*_*i*_) diagram when coupled to a single-mode GRIN fiber is shown in Fig. 2b, which further reveal an optimal pulse energy of 28–30 nJ and a small loss variation of less than 0.2 dB at different depths for a constant pulse energy. Moreover, we illustrate that the high circular symmetry of these waveguide cross-sections results in extremely high mode circularity (see Supplementary text S7 for definition) of greater than 97.0% and as high as 99.5% (Fig. 2c), and the mode circularity varies by ~2% with respect to the writing pulse energy when the measurement error is within 0.2%. It is also worth noting that all four types of waveguides have circularly symmetric Gaussian modes (inset in Fig. 2c), but their mode diameters vary significantly from 8 to 18 μm (Fig. S9) due to the designed differences of RI distributions.

Index grading is an ingenious method for reducing modal dispersion and radiation loss by confining mode energy in the center^26^. Tighter waveguide bends can therefore be achieved in the GRIN waveguide, as shown in Fig. 2d, where a very tight bend radius of 20 mm is demonstrated in GRIN waveguides at 1 dB/cm loss, compared with that larger than 50 mm of other waveguides. In addition, a propagation loss (*L*_*p*_) of 0.2 dB/cm is also revealed in the GRIN waveguide, setting a low coupling loss (*L*_*c*_) of 0.045 dB/facet as *L*_*c*_ = *(L*_*i*_ − *L*_*p*_*)*/2^27^. Thus, it is reasonable to infer that the extremely low loss reported here is due to the excellent mode matching of GRIN waveguide and GRIN fiber such that *L*_*c*_ is minimized. Theoretically, based on the Gaussian field approximation, the *L*_*c*_ between a single-mode fiber and a single-mode waveguide can be calculated as

1 $${L}_{c}\left({dB}\right)\approx -10{\log }_{10}\left(\frac{4{a}^{2}{R}^{2}C}{\left({a}^{2}+{R}^{2}\right)\left({a}^{2}+{R}^{2}{C}^{2}\right)}\right)$$

where *a* is the fiber mode field radius, *R* is the mode field radius (MFD/2) of the waveguide mode in the horizontal direction and *C* is the circularity of the mode field defined by the ratio of the radius in the minor axis direction to that in the major axis direction with a value range of 0–1. As result shown in Fig. S10a, when the diameter and circularity of the waveguide mode field deviate from that of the coupling fiber, the *L*_*c*_ increases logarithmically. At the same time, the mode circularity determines the theoretical lower limit of the *L*_*c*_, that is, the lower the mode circularity, the larger the minimum *L*_*c*_, and the greater the optimal mode diameter than the fiber mode diameter (Fig. S10b). In summary, the developed OCMS method can not only tailor the waveguide size and RI distribution to make the MFD match with a specific fiber but also precisely control the mode circularity to achieve circularity matching, thus enabling lossless interconnection with various single-mode and few-mode fibers.

### Robust and broadband coupling for mode-selective 3D waveguide coupler

PLC-based integrated optics are technically based on 2D planar rectangular or ridge waveguides^28,29^, which have an inherent limitation in propagating and transforming circularly symmetric modes unless an additional lossy mode rotator is inserted^30^. In contrast, 3D waveguides can overcome this limitation by providing new degrees of freedom in controlling optical coupling, including customized cross-sectional shape, controllable RI contrast, as well as 3D spatial geometric arrangements, thereby resulting in a reduced device complexity and an increased compactness. However, the ever-reported 3D waveguide circuits such as in polymers^5,31–33^ and in porous silicon^34^ generally suffer from high losses (*L*_*i*_ ~ 10 dB) and complicated manufacturing processes. In comparison, the FLDW 3D waveguide in glass has lower loss and simpler processing steps. However, in current FLDW techniques, the absence of precise RI profile control method prevents it from being applied for producing high-performance optical coupling devices that are favorably comparable with conventional PLC-based ones. Namely, the mode-coupling structures based on FLDW waveguides, such as mode-selective directional coupler (DC)^35–38^, tapered velocity coupler^39^ and Y-splitters^40–42^, are yet to be optimized in terms of coupling ratios, mode purity and fabrication robustness^43^.

**Fig. 3:**
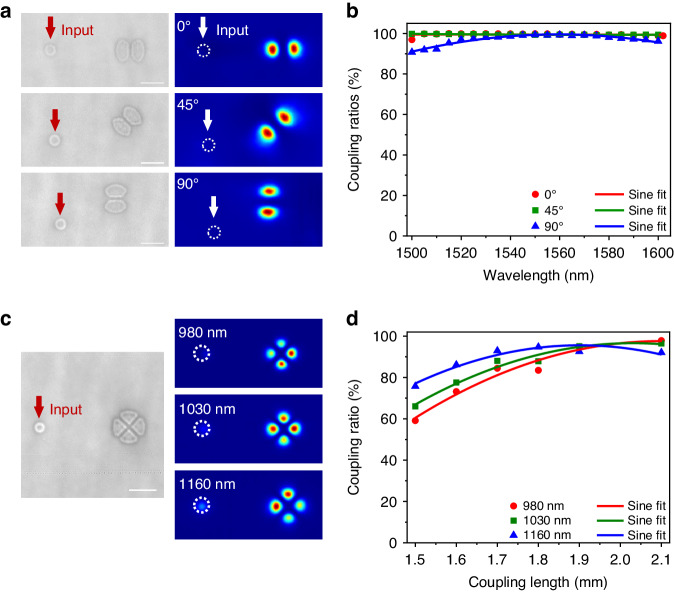
Demonstration of 3D mode-selective waveguide coupler. **a** Optical micrographs (left), measured mode-field distributions (right) of horizontal (0°), angular (45°) and vertical LP_11_-mode DC (90°). **b** The coupling ratios of three LP_11_-mode DCs measured at a broadband of 1500–1610 nm. **c** Optical micrographs (left), measured mode-field distributions (right) of the LP_21_-mode DC at typical wavelengths of 980, 1030 and 1160 nm. **d** The coupling ratio of LP_21_ mode DC as the coupling length increases from 1.5 mm to 2.1 mm. Scale bars are 20 μm

Here, we demonstrate the precise control of mode coupling by the OCMS approach. Firstly, the high mode circularity of OCMS waveguides means that the optical coupling should be spatially isotropic, and the resulting mode-coupled devices should also behave uniformly in 3D space. To illustrate the spatially isotropic coupling, a 3D waveguide coupler for mode-conversion of rotationally asymmetric LP modes, consisting of a direction coupler (DC) structure with a LP_01_-mode core (Core 1) around a LP_11_-mode core (Core 2). Three LP_11_-mode DCs, namely horizontal DC (0°), angular DC (45°) and vertical DC (90°), are precisely geometrically arranged at respective azimuth angles of 0°, 45° and 90° (Fig. 3a), which is theoretically essential for multiplexing all orientation states of rotationally asymmetric LP modes^44^. The designation of an individual LP_11_-mode DC is based on mode coupling theory and using the tried and tested finite element method, where the core diameters are chosen as 8.8 and 23.2 μm, such that the effective RI of the fundamental LP_01_ modes in Core 1 match those of the respective LP_11_ modes in Core 2 over broadband covering 1510, 1550, and 1600 nm (see Supplementary text S9 for more details). The coupling lengths are experimentally chosen to be 0.2 mm to achieve the maximum mode coupling ratio, making a total device length of 20 mm (Fig. S12). Whilst theoretically determining the phase matching conditions and experimentally optimizing the coupling length, the power injected to LP_01_ modes in Core 1 can fully transfer to corresponding LP_11_ modes in Core 2. As shown in Fig. 3b, the device exhibits high mode coupling ratios of 96.9–99.9, 99.2–99.8 and 90.8–99.1% over a broadband of 1500–1610 nm encompassed in the S, C and L telecommunication bands when launched into different LP_01_-mode cores at the azimuth angle of 0°, 45°, and 90°, respectively. Therefore, the OCMS waveguide-based DC performs uniformly over a broadband of 110 nm centered at 1550 nm, with the largest deviation <0.1 dB in coupling ratios. Principally, the broadband performance is primarily due to the inherent characteristics of low dispersion of glass materials in the near infrared band, that is, when the wavelength increases from 1500 nm to 1610 nm, the RI variation is less than 3 × 10^−4^ (see Supplementary text S8 for details). Furthermore, the designed LP_11_ waveguide can break the orientation-degeneracy of LP_11_ modes, enabling high-purity conversion of LP_11_ mode with arbitrary orientations. The measured mode-extinction-ratio (MER) is >25 dB over a broadband of 1500–1610 nm and is largely independent of wavelength (see Supplementary text S10 for details), which outperforms even state-of-the-art mode converters using PLC-based coupler^30^, fused fiber^45^ or 3D polymer waveguides^5,31–33^. The high-purity mode conversion considerably benefits from the mode-selective capability of the mode-shaped cross-section. Theoretically, the mode calculation results (Fig. S12a) reveal that the LP_11_ waveguide does not support the Gaussian distributed fundamental mode, which not only suppresses the propagation of the LP_01_ mode along the waveguide, but also avoids the coupling of the LP_01_ mode between Core 1 and Core 2, thereby maximizing the achieved LP_11_ mode purity and reducing the mode-dependent loss. Furthermore, when injecting Core 1 with a 1550 nm single mode fiber, low losses of 0.6–0.7 dB are achieved. Notably, the device behaves uniformly in changing orientation states of rotationally asymmetric LP modes, thanks to the high circularity of mode field distribution and accurate 3D geometric arrangement of DC structures. In addition, the fabrication robustness of OCMS waveguides enables the accurate geometric arrangement to meet the requirement of precise phase matching in DC structures, avoiding unwanted mode rotation and coupling ratio degradation caused by day-to-day variations of processing parameters in conventional FLDW techniques^43^.

Moreover, the operating mode and wavelength of OCMS-based mode coupler can be easily extended to higher order LP_21_ mode and near-infrared band (Fig. 3c). Similarly, the core diameters of the LP_21_-mode DC are numerically determined as 7.2 and 21.6 μm, such that the effective RI of the fundamental LP_01_ mode in Core 1 match those of the LP_21_ mode in Core 2 over a broadband covering 980, 1030, and 1160 nm (as detailed in Supplementary text S11). The coupling lengths are experimentally chosen to be 1.95 mm to achieve a uniform coupling ratio of 93.0–95.0% (Fig. 3d). The resulting MER is >24 dB (Fig. S11), also indicating a high-purity LP_21_ mode conversion. Thereby, 3D mode-coupling structures based on OCMS waveguides in glass are insensitive to operating wavelengths and structure features, can achieve broadband and robust functions, and should play an important role in the construction of optical routing and integration for next-generation 3D photonic circuits. Notably, OCMS method does not contribute to the broad working bandwidth of above 3D waveguide devices. Instead, the broadband response results from two factors, (1) the low dispersion of the glass material in the near-infrared band, and (2) the 3D waveguide operates in a weak coupling scheme with a coupling length on the millimeter scale. Additionally, this approach of using OCMS waveguides to construct a 3D mode-selective device is also attractive for photonic wire bonding of various-shaped waveguides supporting different Gaussian modes including Hermite–Gaussian modes in Si- or GaAs-based waveguides and Laguerre–Gaussian modes in fibers, thus holds great promise for fabricating hybrid 3D photonic circuits^46^.

### Chip-scaled mode manipulation of ultrashort laser pulses

**Fig. 4:**
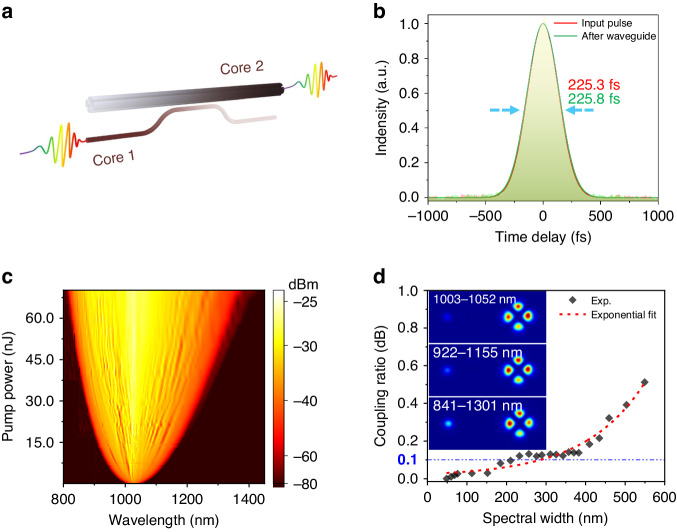
Spatial mode conversion of ultrashort laser pulses. **a** Schematic diagram of mode conversion from an ultrashort LP_01_ mode laser pulse (Core 1) to an LP_21_ mode pulse (Core 2) with negligible time-domain distortion. **b** The pulse intensity distribution measured by the autocorrelator before entering the waveguide and after propagating through the waveguide. **c** Supercontinuum spectra measured at pump power of 1–70 nJ. **d** Measured coupling ratio of Core-2 as a function of the spectral width of the input supercontinuum for directional coupling, where the 0.1 dB deviation in coupling ratios is marked (Inset: measured mode field distributions)

Using the proposed RI engineering tool, we have broken through the technical bottleneck of 3D waveguide mode control and successfully developed a miniaturization platform of OCMS waveguides, which shows great versatilities in terms of ultra-broadband transparency, high processing robustness, as well as high compatibility with fiber-optic systems. In this section, we further demonstrate its prospect in transmitting and manipulating nonlinear laser light by coupling ultrashort laser pulses into the OCMS waveguide (Fig. 4a). A laser pulse with a duration of 225.3 fs (1030 nm, <250 pJ) is coupled with a microscope objective into a waveguide mode converter with a total length of 30 mm (Fig. S19a). In principle, the temporal and spectral characteristics of the input laser pulse are affected not only by the dispersion properties but also by various nonlinear effects of the waveguide. When the input pulse energy is lower than ~250 pJ, the output pulse is broadened by 0.5 fs in the time domain after propagating through the waveguide **(**Fig. 4b), while its pulse shape and spectral width remain unchanged (Fig. S19c). This pulse broadening is believed to be caused by the second-order dispersion of the glass itself, with a calculated critical pulse width of ~58.4 fs and the group delay dispersion of ~1230 fs^2^ for this OCMS-waveguide-based DC (see Supplementary text S12 for more details). Therefore, ultrashort pulses can be conformally transmitted through the OCMS waveguide with negligible distortion in the time domain.

Pumping with intense laser pulses, similar to optical fibers^47^, the incident pulse coupled to the waveguide is under the comprehensive modulation combining dispersion and various nonlinear effects including self-phase modulation, cross-phase modulation, four-wave mixing, and stimulated Raman scattering, leading to the broadening of the spectrum to both short and long wavelengths, and resulting in an extreme spectral broadening referred to as supercontinuum generation. In order to enhance nonlinear effects and generate a strong supercontinuum signal, a 2-m-long optical fiber is connected in front of the waveguide (Fig. S19b), enabling a stable supercontinuum generation and coupling into the DC structure. As shown in Fig. 4c, as the pulse energy increases to the nanojoules level (1–70 nJ), the pulse spectrum broadens dramatically such that the bandwidth exceeds 550 nm. The supercontinuum light input from Core-1 couples to Core-2 for the OCMS-waveguide-based DC over a 210 nm (931–1141 nm) broadband with the largest deviation <0.1 dB in coupling ratios (Fig. 4d). The coupling ratio deviation slowly increases to 0.5 dB as the spectral width broadened to 550 nm (817–1367 nm), while remaining a high-purity mode conversion with an MER higher than 20 dB for LP_21_ mode (Fig. S20). Thus, we have demonstrated a OCMS waveguide device to route fundamental mode, broadband supercontinuum pulse to a desired higher-order-mode one with negligible spatiotemporal distortion. Remarkably, this device outperforms even state-of-the-art 2D planar waveguide coupling devices based on silicon^48–56^, silicon nitride^57–62^, and lithium niobate^63–66^ (Supplementary text S13 and Table S1), with reduced coupling ratio variation by an order of magnitude to 0.1 dB over a broadband covering visible and near-infrared ranges; and the OCMS approach provides a route to achieve ultra-broadband and low-dispersion coupling based on 3D glass waveguides, which has overwhelming advantages in transmitting and manipulating linear and nonlinear laser light over conventional planar waveguide-optic platforms, representing a significant step towards realizing large-scale 3D photonic circuits with great potential to address the scaling challenges in cutting-edge physical applications such as quantum information^67,68^ and 3D optical topology^69–71^.

## Discussion

In summary, we propose a general waveguide fabrication strategy of OCMS, which for the first time allows customizing both RI distribution and cross-sectional morphology of 3D waveguides at the sub-diffraction-limited spatial resolution. Based on the developed OCMS method, we design several centrosymmetric waveguides by tailoring RI profiles as step and gradient distributions, exhibiting single-mode operation with tunable mode diameters ranging from 8 to 18 μm and high mode circularity reaching ~99.5%, achieving record low coupling and insertion losses of 0.045 dB/facet and 0.29 dB, respectively. Moreover, we also demonstrate low-loss 3D waveguide mode-selective couplers by designing corresponding mode-shaped waveguides, reporting non-orientation-degenerate LP_11_ mode conversion with coupling ratios up to ~99.5% and high MER of >25 dB in an ultra-broadband from 1500 to 1610 nm, and also high-purity conversion of LP_21_ mode over a broadband of 980–1160 nm. The OCMS-based device behaves uniformly across varying spatial orientation states of rotationally asymmetric LP modes, thanks to the high circularity of mode field distribution and accurate 3D geometric arrangement of DC structures. Based on these results, we further report an OCMS waveguide device that conformally routes ultrashort pulses and broadband supercontinuum in the time domain, and transforms to a higher-order mode in the spatial domain over a 210 nm broadband with the largest deviation of <0.1 dB in coupling ratios. Additionally, the proposed OCMS method is based on a special regime that confines the heat accumulation effect within the laser focus at low applied pulse energies well below the damage threshold, and is applicable to various glasses regardless of their composition, thus making it a robust and versatile method to integrate 3D waveguide optics into various glass panels. Based on the developed OCMS method, we also delineate an effective way to achieve ultra-broadband, low-dispersion mode conversion of nonlinear laser light, with each mode control unit operating at a bandwidth greater than 210 nm and centered wavelengths covering visible and near-infrared spectrum. Thereby, the OCMS approach demonstrates the unique advantages of 3D glass chips in delivering and manipulating linear and nonlinear laser light with ultra-broadband and represents a remarkable advance in the miniaturization of benchtop optical systems into chip-scale devices.

## Materials and methods

Waveguides are written in the typical commercial Eagle XG glass using a high-repetition-rate chirped pulse amplified femtosecond Yb:KGW laser source (Pharos, Light Conversion) that delivers 226 fs pulses with 1 MHz repetition rate at a central wavelength 1030 nm, and pulse-to-pulse energy keeps stable with a deviation of less than 0.5% RMS over 24 h. A Nikon UPLAN 100× oil-immersed microscope objective (NA = 1.30) is adopted to focus the laser beam at a depth of 20–170 μm. The laser pulse energy deposited in the glasses is finely controlled by the continuous attenuator varying from 10 to 150 μJ. The pulse energy is measured before the objective, and about 60% of the pulse energy is deposited on the sample. All samples are translated by a set of 3-axis computer-controlled high precision Aerotech air-bearing linear stages (ABL1000 and ANT130V-5) with constant scanning speeds of 5 mm/s. It is worth noting that the spatial resolution of the current laser writing strategy is mainly limited by the repetitive positioning accuracy of the linear stages, which in our system is ±50 nm at a maximum travel of 100 mm. The refractive indices of the Corning Eagle XG glass at 254–2066 nm (Table S2) are determined by the experimental data measured by an ellipsometer (UVISEL, HORIBA).

The insertion losses are measured by butt-connect single mode fibers (Corning SMF-28) at two ends of the waveguide, the connection positions are immersed in index-matching oil. Propagation losses of curved single-mode waveguides are determined using the cut-back method by measuring the insertion loss of waveguides of different lengths (10, 30, 50 mm) under the same conditions^72,73^. The mode field distributions are measured by a near-infrared beam profiler (CinCam CMOS camera). The coupling ratio is determined by the ratio of the integral intensities of the fundamental mode waveguide and the higher-order-mode waveguide. The mode extinction ratio, defined as the power ratio of the fundamental mode and LP_11_ mode, is numerically calculated from the mode field distribution using the mode decomposition method^74^.

To study the laser-induced refractive index change, a micro-Raman spectroscopy (Renishaw InVia confocal Raman spectroscope) with 532 nm laser excitation using a ×100 objective operated in confocal mode to achieve a spatial resolution of ~0.5 μm is used to study glass structural changes in laser-irradiated regions and to map refractive index profiles in waveguide cross-sections, by taking into account the correlation between structural modification and RI change (see Supplementary text S4 for more details).

### Supplementary information

Supplementary Information
