# Supplementary material for: Precise mode control of laser-written waveguides for broadband, low-dispersion 3D integrated optics
Source: Light Sci Appl. 2024 Jun 4;13:130. doi: 10.1038/s41377-024-01473-7 (PMC11150431; doi:10.1038/s41377-024-01473-7)
Supplement: Supplementary file 1 — Supplementary Information [file 41377_2024_1473_MOESM1_ESM.docx]

**Supplementary Information for**

**Precise mode control of laser-written waveguides for broadband, low-dispersion 3D integrated optics**

Yuying Wang^1,†^, Lijing Zhong^2,†,*^, Kuen Yao Lau^1^, Xuhu Han^1^, Yi Yang^1^, Jiacheng Hu^1^, Sergei Firstov^3^, Zhi Chen^4,5,*^, Zhijun Ma^4,*^, Limin Tong^1^, Kin Seng Chiang^6^, Dezhi Tan^4,7,*^, Jianrong Qiu^1,2,*^

*^1^College of Optical Science and Engineering, Zhejiang University, Hangzhou 310027, China.*

^2^*Institute of Light+X Science and Technology, College of Information Science and Engineering, Ningbo University, Ningbo, 315211, China*

*^3^Prokhorov General Physics Institute of the Russian Academy of Sciences, Dianov Fiber Optics Research Center, 38 Vavilov str., Moscow 119333, Russia.*

*^4^Zhejiang Lab, Hangzhou, 311121, China.*

*^5^College of Materials Science and Engineering, Key Laboratory of Advanced Materials of Yunnan Province, Kunming University of Science and Technology, Kunming, Yunnan, 650093, China*

*^6^Department of Electrical Engineering, City University of Hong Kong, 83 Tat Chee Avenue, Kowloon, Hong Kong SAR, China.*

*^7^School of Materials Science and Engineering, Zhejiang University, Hangzhou, 310027, China.*

† these authors contributed equally.

* To whom correspondence should be addressed: [zhonglijing@nbu.edu.cn](mailto:zhonglijing@nbu.edu.cn) (L. Z.); [zhichen@zhejianglab.edu.cn](mailto:zhichen@zhejianglab.edu.cn) (Z. C.); [zhijma@zhejianglab.com](mailto:zhijma@zhejianglab.com) (Z. M.); [wctdz@zju.edu.cn](mailto:wctdz@zju.edu.cn) (D. T.); [qjr@zju.edu.cn](mailto:qjr@zju.edu.cn) (J. Q.)

**Contents**

Supplementary text S1: Single scanline at sub-diffraction-limited size in OCMS method: the processing window.

Supplementary text S2: Overlap ratio definition and overlap-controlled multi-scan (OCMS) method.

Supplementary text S3: Positive and negative RI modification in K9 glass by OCMS method.

Supplementary text S4: Measurement of refractive index change by Raman mapping method.

Supplementary text S5: Determination of refractive index change by Inverse-Helmholtz technique.

Supplementary text S6: Mode selection in OCMS waveguides with variable cross-sections

Supplementary text S7: Definition of mode circularity.

Supplementary text S8: Corning Eagle XG glass refractive index measurement.

Supplementary text S9: Design of 3D directional coupler for LP_11_ modes.

Supplementary text S10: Characterization of the mode-selective 3D waveguide coupler.

Supplementary text S11: Design and characterization of 3D directional coupler for LP_21_ modes.

Supplementary text S12: Measured pulse autocorrelation after an objective and after a 30 mm length waveguide.

Supplementary text S13: Comparison of the OCMS-waveguide-based coupling structure with others.

Table S1. Comparison of OCMS-waveguide-based coupling structure based on glass 3D waveguides with others.

Table S2. Corning Eagle XG glass refractive indices data.

## Supplementary text S1: Single scanline at sub-diffraction-limited size in OCMS method: the processing window.

Typically, FLDWs operating in a thermal regime with a high repetition rate (e.g., >200 kHz) induce localized RI change upon thermal-accumulation effects^1^, resulting in a common waveguide structure consisting of a central heat-accumulation core and a surrounding thermal diffusion cladding^2^. The thermal diffusion region can create a complicated RI structure in the glass and greatly expand the diameter of the waveguide, making it difficult to build precisely controlled RI structures from a simple stack of multi-scans, resulting in rough control over the waveguide cross-sectional morphology. Therefore, it is crucial to suppress the thermal diffusion effect during the FLDW process in order to achieve fine control over the scanline diameter and realize high spatial resolution when configuring waveguide through multi-scan method. From the experimental result in **Fig. S2**, the threshold pulse energy required to induce a microscopically observable modification inside the glass is 20 nJ measured in front of the objective, and corresponding optical breakdown threshold power (intensity) is ~0.75 MW (~7.5×10^14^ W m^-2^) by taking account of the 60% transmittance of the objective. We apply low pulse energy ranging from 20 to 30 nJ for OCMS waveguide fabrication, 1.0-1.5 times greater than the optical breakdown threshold but well below the self-focusing threshold^3, 4^, where the shape and size of the modified region is determined by the focal volume of the target. In other words, it is free of thermal diffusion and the filamentation effect and thus the laser-induced modification is localized within the focal region. Additionally, the absence of self-focusing avoids focus position shift due to self-phase modulation and self-focusing effects and is beneficial to achieve high spatial positioning accuracy, which is crucial for finely manufacturing waveguide coupling structures to achieve a high coupling ratio.

The critical threshold power for self-focusing *P_cr_* can be estimated as:

$$P_{cr}=\frac{3.77\lambda^{2}}{8\pi n_{0}n_{2}}$$

where 3.77 is the coefficient for the Gaussian beam, 𝜆=1030 nm is the central wavelength of laser, with *n_0_* =1.453 being the linear refraction index and *n_2_* = 3.54×10^-16^ cm^2^ W^-1^ the nonlinear refraction index for fused silica^5^. For fused silica glass, *P_cr_* is ~3.09 MW. Compared to fused silica glass, multi-component silicate glasses (e.g. Eagle XG and Gorilla-3 glasses) have a lower self-focusing threshold because their bandgap is only about one-third that of fused silica. In general, thermomechanical properties can be strongly altered by the presence of network modifiers, they relax the strength of covalent bonds to produce less rigid bonds and favor expansion and rarefaction in response to laser radiation. However, multi-component silicate glass has a more pronounced thermal diffusion effect, with an order of magnitude higher coefficient of thermal expansion than fused silica glass, which is favoring for the heat accumulation effect and is critical for forming a uniform RI modification.


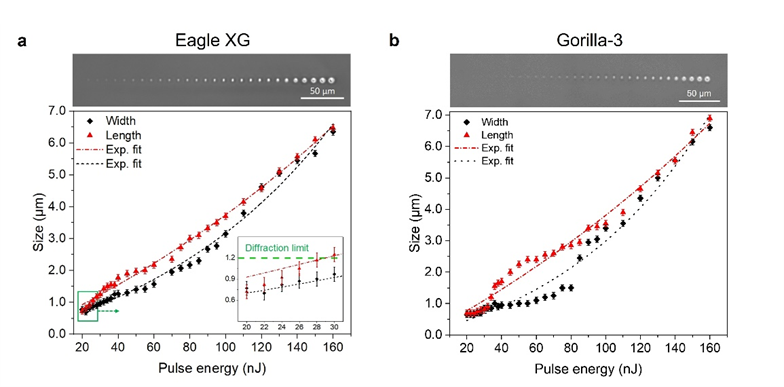


**Supplementary Fig. S1** (Upper) The morphological evolution of the cross-section of waveguides written in Eagle XG (**a**) and Gorilla-3 (**b**) with increased pulse energy from 20 to 160 nJ. (Down) Dependence of waveguide size (width and length) on applied pulse energy with an enlarged view (**a**, inset) shows the processing window of a single trace size smaller than the diffraction limit of the objective (100X, 1.30 NA).

## Supplementary Text S2: Overlap ratio definition and overlap-controlled multi-scan (OCMS) method.

The overlap ratio (*OR*) in the radial and azimuthal directions is defined by the ratio of single scanline diameter (*D=*800 nm) to adjacent scanlines spacing (*L*): OR *= D/L*. The OR is equal in the azimuthal direction and varies in the radial direction, and every two consecutive scanlines are inscribed symmetrically about the center of the waveguide. For the step-like (I) waveguide, the overlap of adjacent tracks is the same (i.e. OR = 1.0) across the waveguide cross-section, making it possible to form a step-index profile. For the GRIN (II) waveguide, the OR is 4.0 in the center and decreases radially to 1.0 where the waveguide meets the pristine glass. For the “W”-like (III) and “U”-like (IV) waveguides, the OR peak of 1.7 is located in the outermost ring. The difference is that the center of the "W" waveguide has an equal OR to the outer ring, while the center of the "U"-like waveguide has an OR of 1.0. When designing the waveguide cross-section, by carefully arranging the scanlines, the OR value at different positions is continuously changed from 1.0 to 16.0 with a precision of 0.1, and the width of the corresponding RI modification unit is controlled between 50 and 800 nm. Notably, every two consecutive scanlines are inscribed symmetrically about the center of the waveguide, and the order of adjacent scanline pairs is arranged from outside to inside in order to avoid asymmetric accumulation of stress^6^.


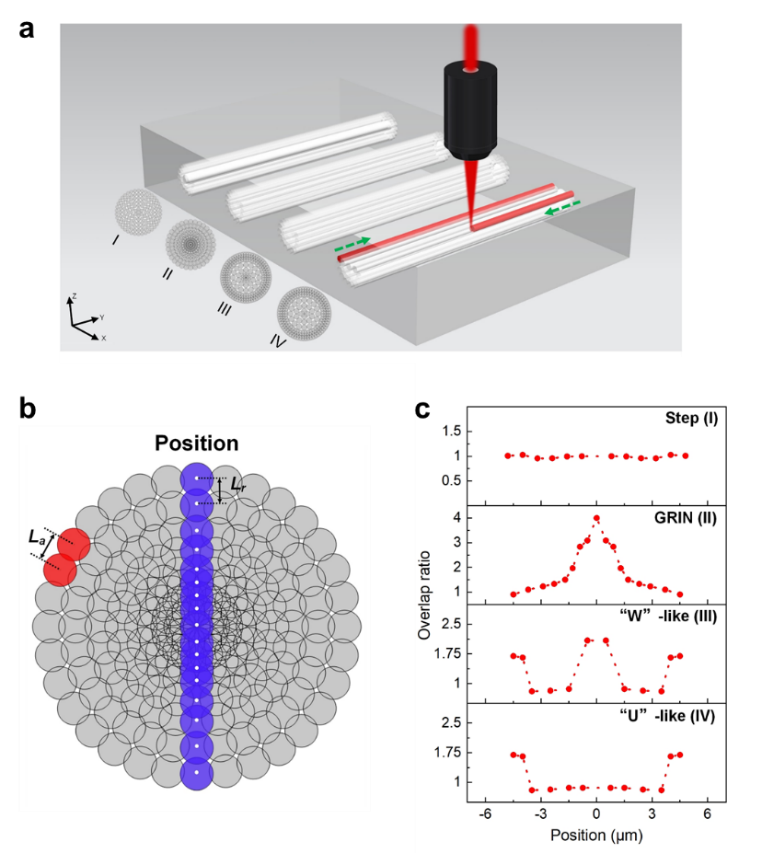


**Supplementary Fig. S2 a,** Schematic illustration of OCMS method. **b,** Azimuthal OR distribution of waveguide cross-sections for four OCMS waveguides. **c,** Multi-scan scheme of the GRIN waveguide, *L_a_* and *L_r_* represent the distance between adjacent scanlines in azimuth and radial directions, respectively.

**Supplementary text S3: Positive and negative** RI **modification in K9 glass by OCMS method.**

K9 glass is considered to be a typical alkali-rich borosilicate glass, and ultrashort laser irradiation usually leads to RI modification, or both positive and negative RI modifications appear in the cross-section^7^. Here, through the developed OCMS method, we achieve all-positive (Fig. S3a) or all-negative (Fig. S3b) RI medications by accurately control the laser pulse energy. The resulting positive RI contrast is small compared with that of Eagle glass, but the negative RI contrast is more remarkable. Generally, Borosilicate glass has a low soften temperature and is widely used as a glass matrix for active-ion doping and nanocrystal precipitation^8^. Therefore, the fine control of the negative refractive index change reported here also implies arbitrarily tailoring the degree of laser-induced thermal modification with extremely high resolution, thus providing an efficient way to generate submicron or nanoscale structural changes in glasses.


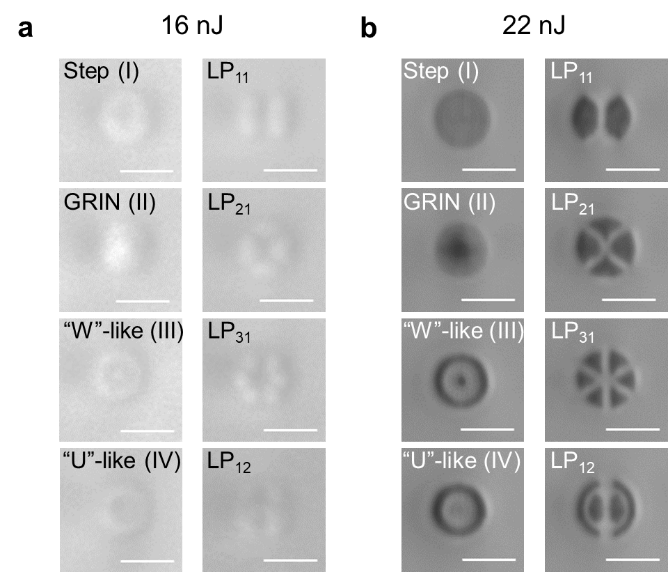


**Supplementary Fig. S3.** Optical micrographs of four fundamental-mode-shaped modification (left), LP_11_, LP_21_, LP_31_ and LP_12_ mode-shaped modification (right) in a K9 glass under a laser pulse energy of 16 nJ (**a**) and 22 nJ (**b**). Scale bars are 10 μm.

## Supplementary text S4: Measurement of refractive index change by Raman mapping method.

Comparison of Raman spectra in the laser-irradiated area and in the pristine glass is generally used to reveal the structural reorganization induced by laser irradiation. In both Eagle XG glass (**Fig. S4**) and FS-7980 glass (**Fig. S5**), the two Raman peaks at about 487 (D_1_) and 604 cm^-1^ (D_2_) are assigned to the symmetrical stretching mode of bridging oxygen in the 4- and 3-membered rings, respectively^9^. In Gorilla-3 glass (**Fig. S6**), D_1_(480 cm^−1^) and D_2_ (580 cm^−1^) peaks correspond to symmetric stretching of O atoms, namely, breathing mode, in the four- and three-membered rings of TO_4_ tetrahedra, respectively, where T represents Al or Si. The A_1_ band (1090-1100 cm^−1^) corresponds to an in-phase stretching of the four O atoms in TO_4_ tetrahedra^10^.

The reason for picking the D_1_, D_2_ and A_1_ peaks is that the intensity difference of these peaks under different laser irradiation conditions can be used as a parameter to determine the degree of structural modification. Typically, the increment of D_1_ and D_2_ peaks is well accepted as origin of glass densification and positive refractive index change in the waveguide^9, 11, 12^. Principally, Raman peak intensity changes are linearly related to laser-induced glass density changes^12^, and then the RI is determined by the glass density through the Clausius-Mossotti relation^13, 14, 15^ or Lorentz–Lorenz equation^16, 17, 18^. Therefore, the Raman mapping method used in this work is based on direct material density measurements, revealing the true RI distribution of the waveguide cross-section. In this work, the Raman mapping method has a high spatial resolution of ~0.5 μm, which is determined by the resolution of the micro-Raman microscope system (see Methods).


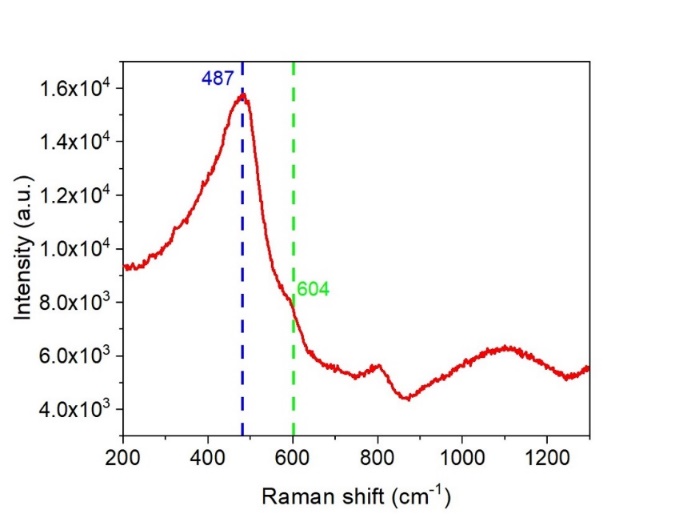


**Supplementary Fig. S4.** Measured Raman spectra of the typical waveguides written in Eagle XG glass with selected peak at 487 cm^-1^ and shoulder at 604 cm^-1^.


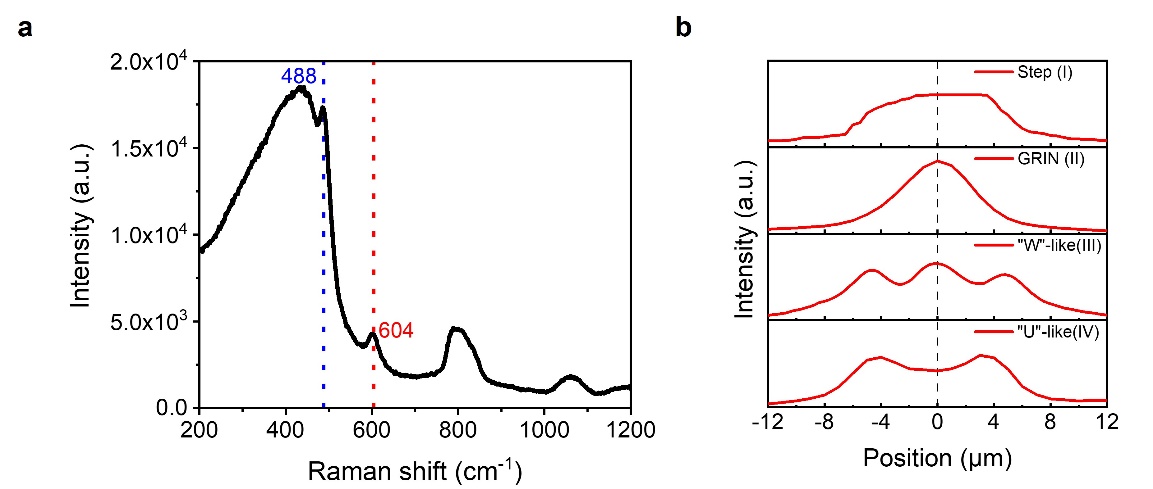


**Supplementary Fig. S5 a,** Measured Raman spectra of the typical waveguides written in FS-7980 glass with selected peak at 487 cm^-1^ and shoulder at 604 cm^-1^. **b,** Measured Raman peak intensity distributions of four types of fundamental-mode waveguides with step (I), GRIN (II), “W”-like (III) and “U”-like (IV) distributions, respectively.


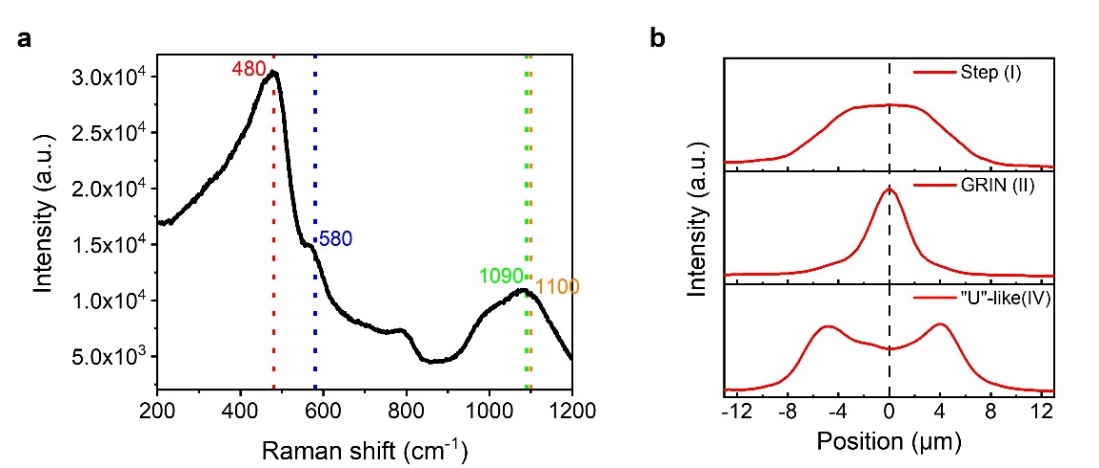


**Supplementary Fig. S6** Measured Raman spectra of the typical waveguides written in Gorilla-3 glass with selected peak at 480 cm^-1^ and shoulder at 580 cm^-1^. Measured Raman peak intensity distributions of three types of fundamental-mode waveguides with step (I), GRIN (II) and “U”-like (IV) distributions, respectively.

## Supplementary text S5: Determination of refractive index change by Inverse-Helmholtz technique.

Although the Raman mapping method reflects the actual RI distribution and has a high spatial resolution, we find it challenging to quantify RI change accurately. Therefore, as a complementary method or comparison, we use a numerical method of Inverse-Helmholtz technique to study the RI change, which is based on the measurement of near-field mode distributions^19, 20^:

$\Delta n(x,y) = \frac{\lambda^{2}}{{4\pi}^{2}n_{0}}\frac{\nabla^{2}E(x,y)}{E(x,y)}$ (1)

where, λ is the laser wavelength, and *n_0_* is the refractive index of substrate glass. The normalized E-field distribution *E(x,y)* can be inferred by the measured near-field mode intensity distribution. In the calculation, a third-order Butterworth filter with optimized cutoff frequency is employed to remove impulsive and high-frequency noise in the measured intensity. As results shown in **Fig. S7(a)**, the near-field method reveals an increase in the RI change from 1.55×10^-3^ to 3.23×10^-3^ as the OR varies from 1.0 in the step-like waveguide to 4.0 in the center of the GRIN waveguide, indicating that the RI change can be accurately controlled with a precision of ~5.6×10^-5^ by changing the OR value. However, in principle, the near-field method only reveals the equivalent RI change, which reflects the overall effect of the RI distribution on the mode field, and cannot distinguish the actual spatial variation of the RI. As shown in **Fig. S7(b-e)**, the obtained RI profiles of the four types of waveguides have similar "core-cladding" shaped distributions except for different RI contrast and diameter. However, by combining these two RI measurement methods of Raman mapping and inverse Helmholtz calculation, we provide a comprehensive glimpse into the control capability of the OCMS method on the waveguide RI profile. Namely, the Raman mapping method shows that the OCMS method constructs the waveguide cross-section with high spatial resolution, while the near-field method reveals that the RI change can be precisely controlled by changing the OR. Therefore, the OCMS provides a precise and accurate method to tailor the RI profile of the FLDW waveguide in glass.


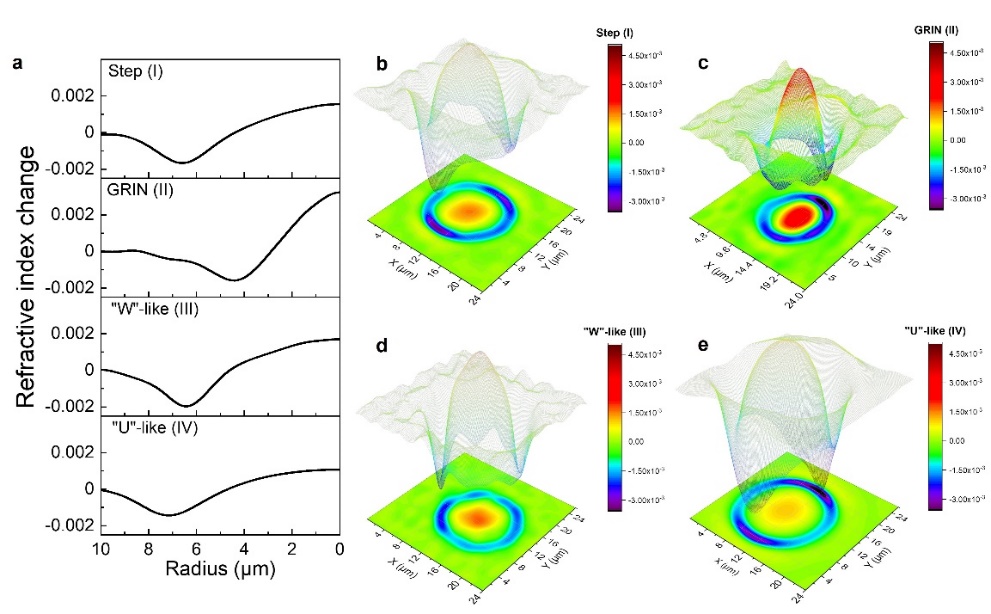


**Supplementary Fig. S7 a,** Refractive index distributions of four types of waveguides in the radial direction using inverse Helmholtz technique. **b-e,** Numerical reconstruction of refractive index profiles of four types of waveguides using the inverse Helmholtz technique.

## Supplementary text S6: Mode selection in OCMS waveguides with variable cross-sections.

Through the OCMS approach, it is supposed to fabricate a waveguide with the mode-selective capability by tailoring the waveguide core to have the same profile as a specific mode field. **Fig. S8** illustrates designed waveguides with centrosymmetric cross-sections for the purpose of selecting on-demand Laguerre-Gaussian modes, where their cross-sectional shape and also RI distribution are made to match the intensity distribution of LP_11_, LP_21_, LP_31_ and LP_12_ modes. The 2D Raman mapping results show a step-index distribution with a shape consistent with designs, indicating that the proposed OCMS method is capable of delineating waveguide cross-sectional shapes with high spatial resolution. The reason for making the mode-shaped cross-section is based on the fact that the insertion of intensity- or phase-modulating elements with the same distribution as the laser mode can efficiently select the corresponding mode while suppressing other un-deserved modes in the beam propagation path^21^, according to the mode competition theory in laser optics^21, 22^. Here, in principle, the null RI change regions, including all un-irradiated regions, especially the center of the waveguide, could lead to a propagation loss of the fundamental mode that is significantly higher than that of the deserved high-order mode, allowing selection of the high-order mode at sufficiently long propagation distances. Experimentally, when these high-order-mode waveguides are directly butt-coupled with a single-mode fiber at 1550 nm, the LP_11_-waveguides with spatial orientation angles of 0°, 45° and 90° can effectively convert the fundamental LP_01_ mode to the corresponding LP_11_ modes on a centimeter length scale with a mode extinction ratio (MER) of ~5 dB. The measured mode field distributions are oriented in the same direction as the waveguide cross-sections, implying a new way that allows excitation and stable transmission of LP_11_ modes of arbitrary orientations. Notably, designed waveguides are free of interference effect between orthogonal modes (such as LP_11a_ and LP_11b_) and mode rotation issues, which could facilitate divergence-degenerate spatial multiplexing application of high-order modes^23^, while these problems are common in optical fibers^24^ and FLDW waveguides^25^. Here, to the best of our knowledge, we are the first to realize high-order-mode waveguides for LP_21_, LP_31_ and LP_12_ modes using FLDW technology inside glass, and these modes can be activated and converted with high mode purity by designing corresponding mode-selective couplers. In addition, the mode-selective capabilities of these high-order mode waveguides also inspire applications for insertion into photonic circuits and fiber laser resonators as mode filters and mode selectors, which share the inherent merit of high damage threshold of glass material over polymer and metal thin films^26, 27^.


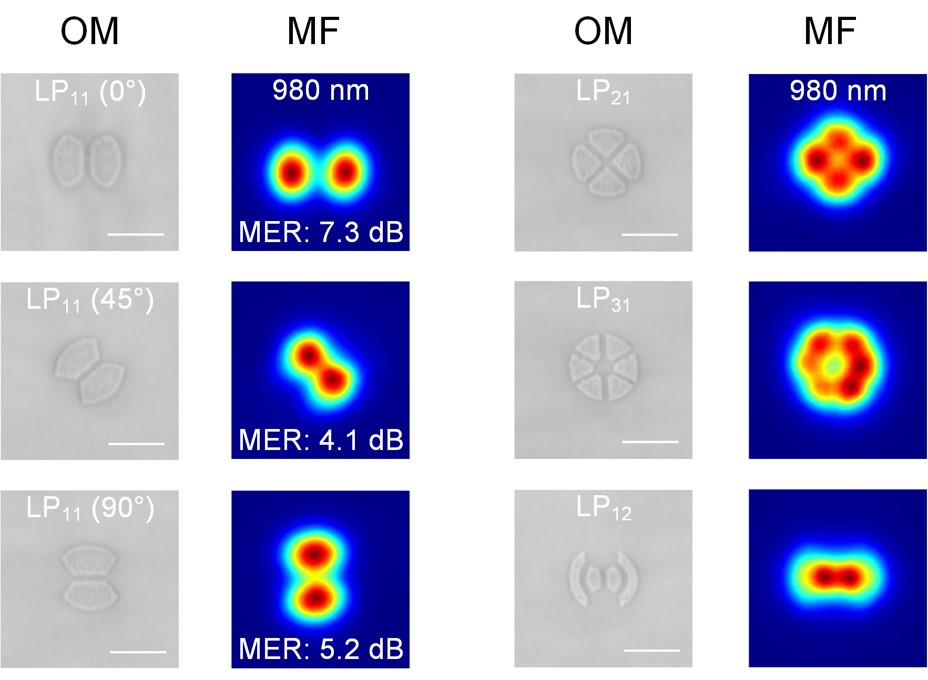


**Supplementary Fig. S8** Optical micrographs (OM) and mode field (MF) distributions of LP_11_(0^o^), LP_11_(45^o^), LP_11_(90^o^), LP_21_, LP_31_ and LP_12_ mode waveguides, respectively. Scale bars are 20 μm.

**Supplementary Fig. S9** Mode field diameter of four types of single-mode waveguides. Mode field diameter of four types of single-mode waveguides written with 26-30 nJ pulse energies and a constant scan speed of 5 mm s^-1^.

**
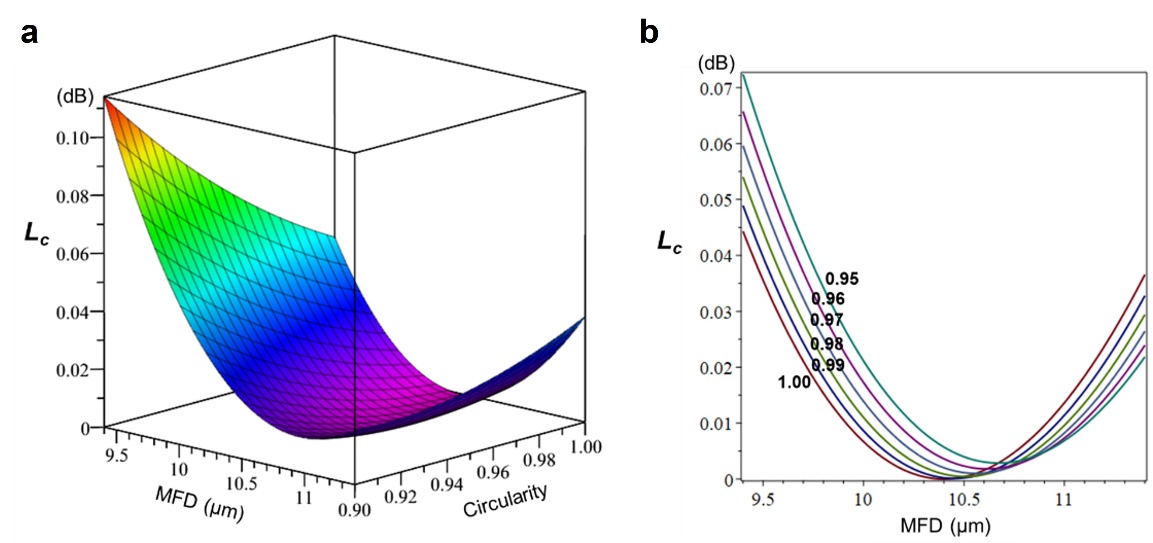
**

**Supplementary Fig. S10** Theoretical calculation of the coupling loss (L_c_). **a,** A 3D plot of the calculated *L_c_* between a single-mode waveguide at 1550 nm and a single-mode fiber with a 10.4 μm MFD. **b**, A 2D plot of the calculated *L_c_* by preset 0.95-1.0 mode circularity.

## Supplementary text S7: Definition of mode circularity.

According to the measured mode-field distribution, the circularity of the waveguide mode can be calculated by:

$e=\frac{d}{D}$ (2)

where e represents the circularity and is equal to the ratio of the short main axis (d, minor) to the long main axis (D, major). For a circular intensity distribution, this value is close to 1. For strongly elliptical distributions, this value is close to 0.

## Supplementary text S8: Corning Eagle XG glass refractive index measurement.

The refractive indices of the Corning Eagle XG glass at 254-2066 nm (**Table S2**) are determined by the experimental data measured by an ellipsometer (UVISEL, HORIBA). The refractive indices at 1510 nm, 1550 nm and 1600 nm are 1.49800, 1.49786 and 1.49773, respectively. When the wavelength increases from 1500 nm to 1610 nm, the RI variation is less than 3×10^-4^. As shown in **Fig. S11**, the refractive index changes gently in the range of 800–2000 nm, which also indicates low dispersion of the glass.

**
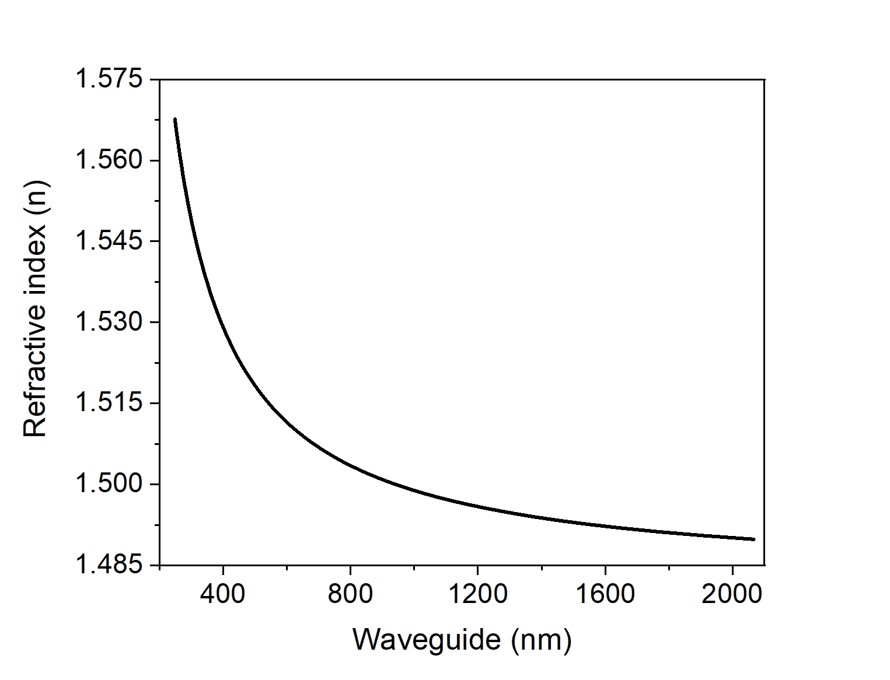
**

**Supplementary Fig. S11** Experimental measurement of the refractive index of Corning Eagle XG glass.

## Supplementary text S9: Design of 3D directional coupler for LP_11_ modes.

The coupled-mode theory for evanescent-field coupling in a weakly guiding structure is used to estimate coupling coefficient between two cores^28^, the LP_01_ mode in Core 1 and LP_11_ mode in Core 2 are designed to be phase-matched, i.e. the two modes have same effective *RI*. We estimate the effective RI of the different modes for Core 1 and Core 2 with the full-vector finite-element method^29^. The top-view of the two-core mode couplers are shown in **Fig. S12a**. At both the input and output of the mode couplers, cosine S-bends with a radius of 100 mm are used to spatially separate the straight sections, the core-to-core spacing and coupling spacing are 71 µm and 21 µm, respectively. As an example, we design the directional coupler using step-like (I) waveguide, hence the two cores have the same *RI*. In the design, the RI profile of the Core 1 is circular symmetrical and Core 2 is non-circular symmetrical. By decreasing the effective RI of the fundamental mode through using a smaller diameter of the Core 1, and increasing the effective RI of LP_11_ mode through using a lager diameter of the Core 2, two cores achieve phase matching. According to the coupled mode theory^30, 31, 32, 33^, the coupling ratio of DCs is closely related to coupling coefficient (*k*), coupling length (*L*), and propagation constant (*β*) by following equation:

$Coupling ratio=\frac{I_{2}}{I_{1}+I_{2}}=\frac{4k^{2}}{4k^{2}+{\Delta\beta}^{2}}\cdot{sin}^{2}(\frac{\sqrt{4k^{2}+{\Delta\beta}^{2}}}{2}\cdot L+\varphi_{0})$ (3)

where, *Δβ=|β_1_-β_2_|* is the difference between the two waveguide propagation constants, and *I*_1_ (*I*_2_) denotes the output power of Core 1 (Core 2). In our cases, φ_0_ is a constant caused by the coupling in the bending regions of DCs.

In the numerical calculation, the RI of the pristine glass is set to that of Corning Eagle XG glass, which is 1.4980, 1.4979 and 1.4977 at three characteristic wavelengths of 1510 nm (S-band), 1550 nm (C-band) and 1600 nm (L-band), respectively. The relative difference in RI between pristine glass and waveguide is 0.002^2^. We calculate the effective RI of Core 1 and dispersion relation shown in **Fig. S12b**. The Core 2 is a hybrid core, which supposes two modes, namely LP_01_ and LP_11_ mode. These two modes are the supermodes of two coupled single-mode waveguide cores^28^. The LP_01_ mode has a higher effective RI of 1.4987 than 1.4982 of the LP_11_ mode at 1550 nm. When the diameters of Core 1 and Core 2 are set to 8.8 and 23.2 μm, the phase-matching condition between the LP_01_ mode of Core 1 and LP_11_ mode of Core 2 are satisfied at a broadband covering 1510 nm, 1550 nm and 1600 nm with the effective RI of 1.4983, 1.4982 and 1.4980, respectively. Notably, the 8.8 μm size of Core 1 is close to the core diameter of 1550 nm single-mode fiber (SMF) of 8.2 μm, which aims to better mode match with the SMF and reduce the insertion loss of DC devices.


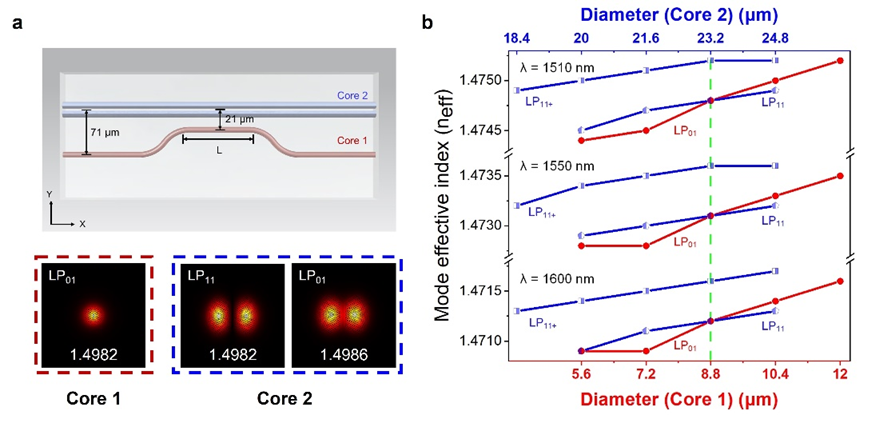


**Supplementary Fig. S12 a,** (Upper) Schematic diagram of DC, which consists of a fundamental mode waveguide (Core 1) and a high-order mode waveguide of LP11 (Core 2); (Down) Mode intensity distributions for Core 1 and Core 2 with diameters of 8.8 µm and 23.2 µm, respectively, where the numbers embedded in the patterns are the effective RI of the corresponding modes. **b,** Variations of the effective indices of LP01 and LP11 modes with the diameters of Core 1 (red squares) and Core 2 (blue triangles) at 1510 nm, 1550 nm, and 1600 nm, respectively, where the green vertical dotted line highlights the chosen diameters for Core 1 (8.8 µm) and Core 2 (23.2 µm) for satisfying the phase-matching condition.

**Supplementary text S10: Characterization of the mode-selective 3D waveguide coupler.**

The mode coupler is characterized by launching light separately into the LP_01_ waveguides and imaging the end facet of the glass chip using a near-infrared beam profiler (CinCam CMOS camera). Typical captured near-field distributions excited from the horizontal LP_01_ waveguide are shown in **Fig. S13a**, **Fig. S14a** and **Fig. S15a**, where a 1550 nm single-mode fiber is used to butt-couple a broadband laser light (Santech TSL-550) into the glass chip with the laser center wavelength continuously tuning from 1500 to 1610 nm. The coupling ratio, which quantifies the total power transfer from the single-mode waveguide to the multimode waveguide, is determined from the ratio of the integrated intensities imaged by the beam profiler (**Fig. S13b-d**, **Fig. S14b-c** and **Fig. S15b-c**). The mode extinction ratio, defined as the power ratio of the LP_01_ and LP_11_ modes, is calculated from the near-field distribution, for which the residual light in the null of the LP_11_ mode is used to estimate the power of the underlying LP_01_ mode^25, 34^. **Fig. S13e**, **Fig. S14d** and **Fig. S15e** show mode extinction ratios measured from horizontal (d), angular (e) and vertical (f) LP_01_ waveguide excitation.

These DCs have different coupling lengths ranging from 0.2 to 5 mm, as well as fixed coupling distances. The input and output sections each consist of two direct waveguides with a spacing of 71 μm, connected to the coupling region by curved waveguides with a bending radius of 100 mm. As shown in **Fig. S13b**, **Fig. S14b** and **Fig. S15b** as examples, by properly selecting the coupling length, the LP_01_ mode is completely converted to the LP_11_ mode output, in the broadband range of 1500-1610 nm, that is, the coupling ratio is largely independent of the wavelength. It can be seen from equation (3) that under a constant coupling distance, as the coupling length increases, the change of coupling ratio conforms to the sine curve (**Fig. S13c**). Coupling coefficient (k) increases from 0.97 to 1.27 rad mm^-1^ as laser power varies from 26 mW (26 nJ) to 30 mW (30 nJ). As shown in **Fig. S13d**, **Fig. S14c** and **Fig. S15c** the wavelength-dependence also closely fits analytic models^35^. These DCs also exhibit high MER of 30.9-44.9 dB (**Fig. S13e**), 28.4-42.7 dB (**Fig. S14d**), 24.3-41.8 dB (**Fig. S15d**).


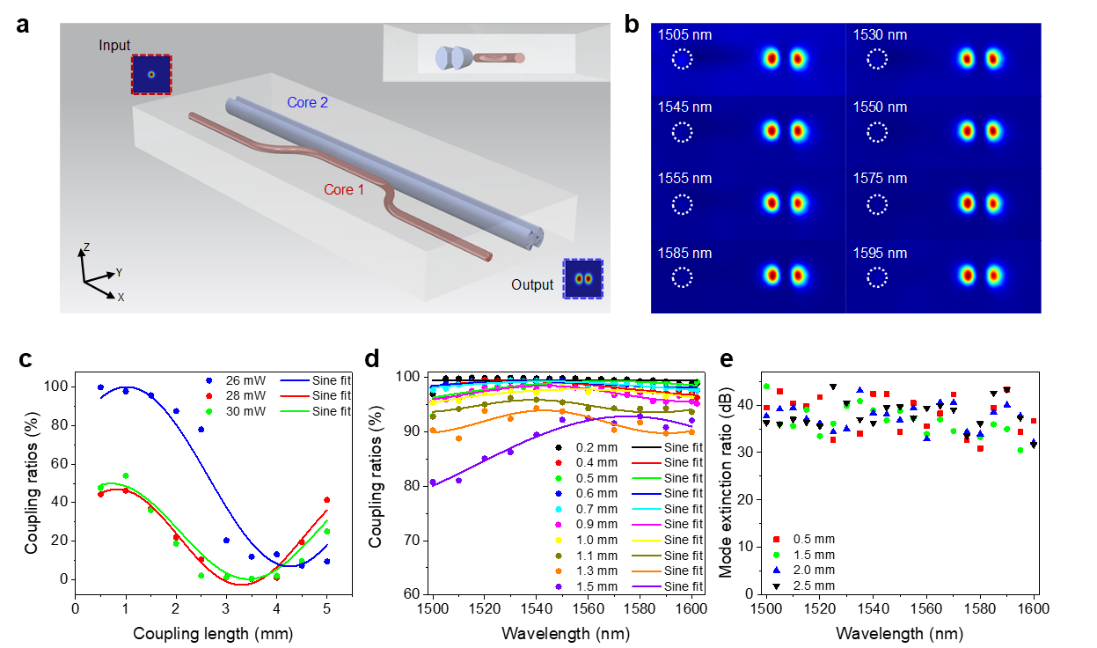


**Supplementary Fig. S13** Horizontal LP_11_-mode directional coupler (0°). **a,** Schematic diagram of LP01 to LP_11_ mode conversion using an DC. **b,** Output near-field distributions excited from the horizontal LP_01_ waveguide as the center wavelength changed from 1500 to 1610 nm. **c,** Measured coupling ratios with various coupling lengths (0.5-5 mm) at a wavelength of 1550 nm. **d,** Measured coupling ratios with the wavelength changed from 1500 to 1610 nm at various coupling lengths (0.2-1.5 mm). **e,** Measured mode extinction ratios of output LP_11_ modes at a coupling length of 0.5-2.5 mm and a wavelength of 1500-1610 nm.


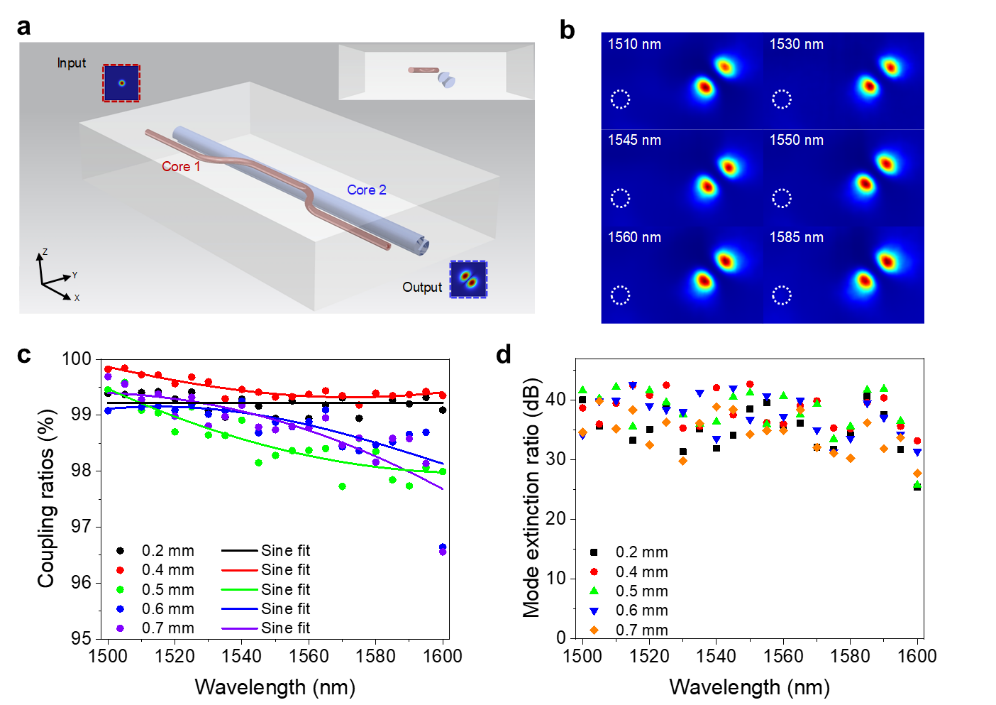


**Supplementary Fig. S14 Angular LP_11_-mode directional coupler (45°)**. **a**, Schematic diagram. **b**, Output near-field distributions excited from the horizontal LP_01_ waveguide as the center wavelength changed from 1500 to 1610 nm. **c**, Measured coupling ratios with the wavelength changed from 1500 to 1610 nm at various coupling lengths (0.2-0.7 mm). **d**, Measured mode extinction ratios of output LP_11_ modes at a coupling length of 0.2-0.7 mm and a wavelength of 1500-1610 nm.


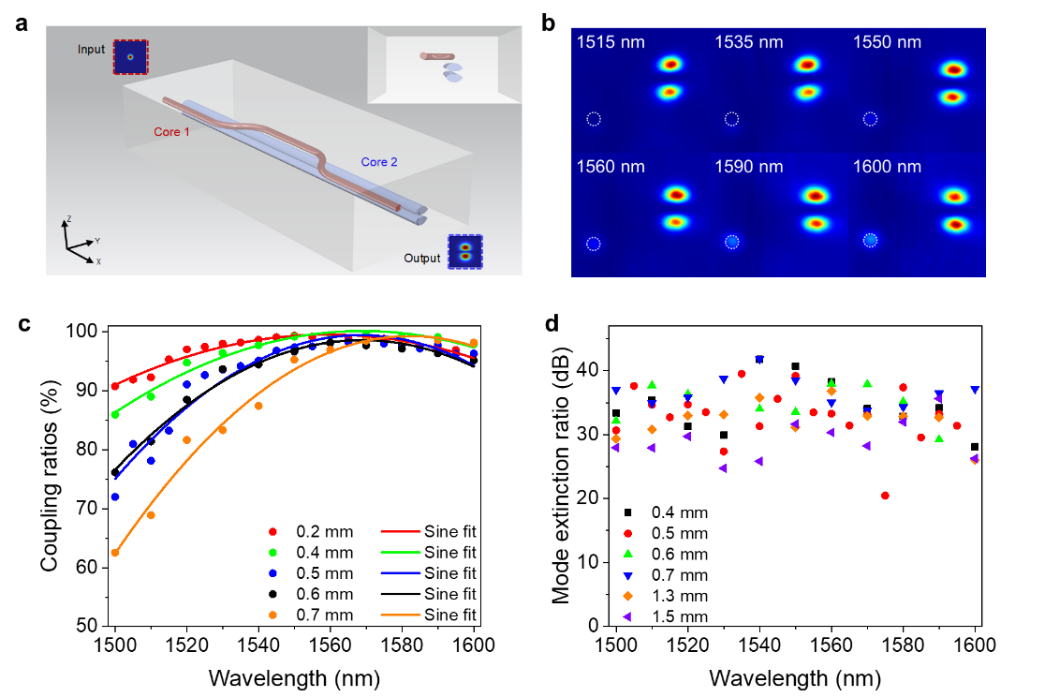


**Supplementary Fig. S15** Vertical LP_11_-mode directional coupler (90°). **a**, Schematic diagram. **b**, Output near-field distributions excited from the horizontal LP_01_ waveguide as the center wavelength changed from 1500 to 1610 nm. **c**, Measured coupling ratios with various coupling lengths (0.2-1.4 mm) at a wavelength of 1550 nm. **d**, Measured coupling ratios with the wavelength changed from 1500 to 1610 nm at various coupling lengths (0.2-0.7 mm). **e**, Measured mode extinction ratios of output LP_11_ modes at a coupling length of 0.4-1.5 mm and a wavelength of 1500-1610 nm.

**Supplementary text S11: Design and characterization of 3D directional coupler for LP21 modes.**

Similar to LP_11_-mode DC as detailed in the **Supplementary text S9**, we design a LP_21_-mode DC (**Fig. S16a**). Cosine S-bends with a radius of 100 mm are used to connect straight sections. The core-to-core spacing and coupling spacing are 69.4 µm and 19.4 µm, respectively. We calculate the effective RI of Core 1 and dispersion relation shown in **Fig. S16b**. The Core 2 is a hybrid core, which supposes two modes, namely LP_01_ and LP_21_ mode. These two modes are the supermodes of two coupled single-mode waveguide cores^28^. The LP_01_ mode has a higher effective RI of 1.5018 than 1.5012 of the LP_21_ mode at 980 nm. When the diameters of Core 1 and Core 2 are set to 7.2 and 21.6 μm, the phase-matching condition between the LP_01_ mode of Core 1 and LP_21_ mode of Core 2 are satisfied at a broadband covering 980 nm, 1030 nm and 1160 nm.

Based on the numerical design, we fabricate LP_21_-mode DCs with coupling length increased from 1.5 mm to 2.2 mm (**Fig. S17**). The mode field distributions (**Fig. S17a-c**), coupling ratios (**Fig. S17d-f**) and MERs (**Fig. S17g-i**) are characterized at different center wavelengths of 980 nm, 1030 nm and 1160 nm, respectively. Notably, a home-made 1160 nm CW laser (**Fig. S18**) is used for the measurement.


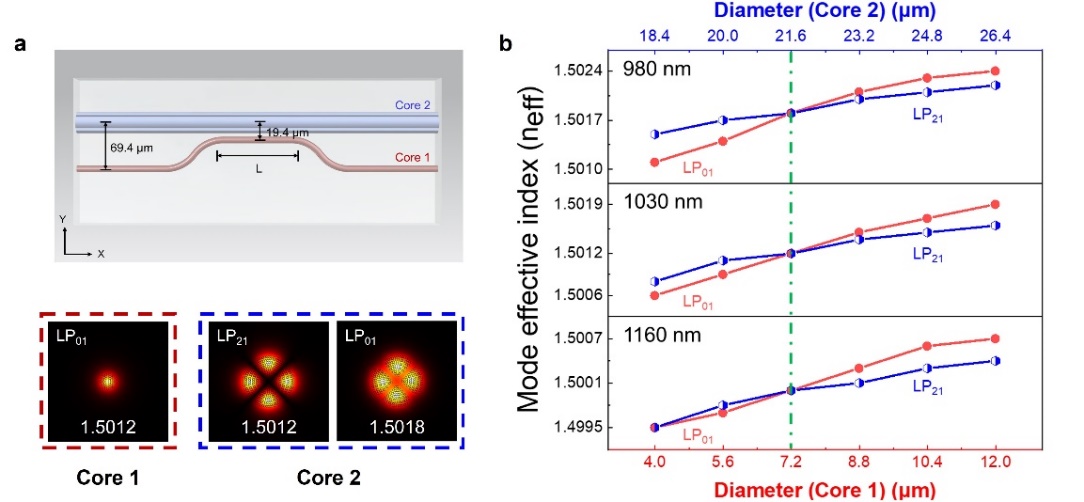


**Supplementary Fig. S16 a**, (Upper) Schematic diagram of DC, which consists of a fundamental mode waveguide (Core 1) and a high-order mode waveguide of LP_11_ (Core 2); (Down) Mode intensity distributions for Core 1 and Core 2 with diameters of 8.8 µm and 23.2 µm, respectively, where the numbers embedded in the patterns are the effective RI of the corresponding modes. **b**, Variations of the effective indices of LP_01_ and LP_11_ modes with the diameters of Core 1 (red squares) and Core 2 (blue triangles) at 1510 nm, 1550 nm, and 1600 nm, respectively, where the green vertical dotted line highlights the chosen diameters for Core 1 (8.8 µm) and Core 2 (23.2 µm) for satisfying the phase-matching condition.


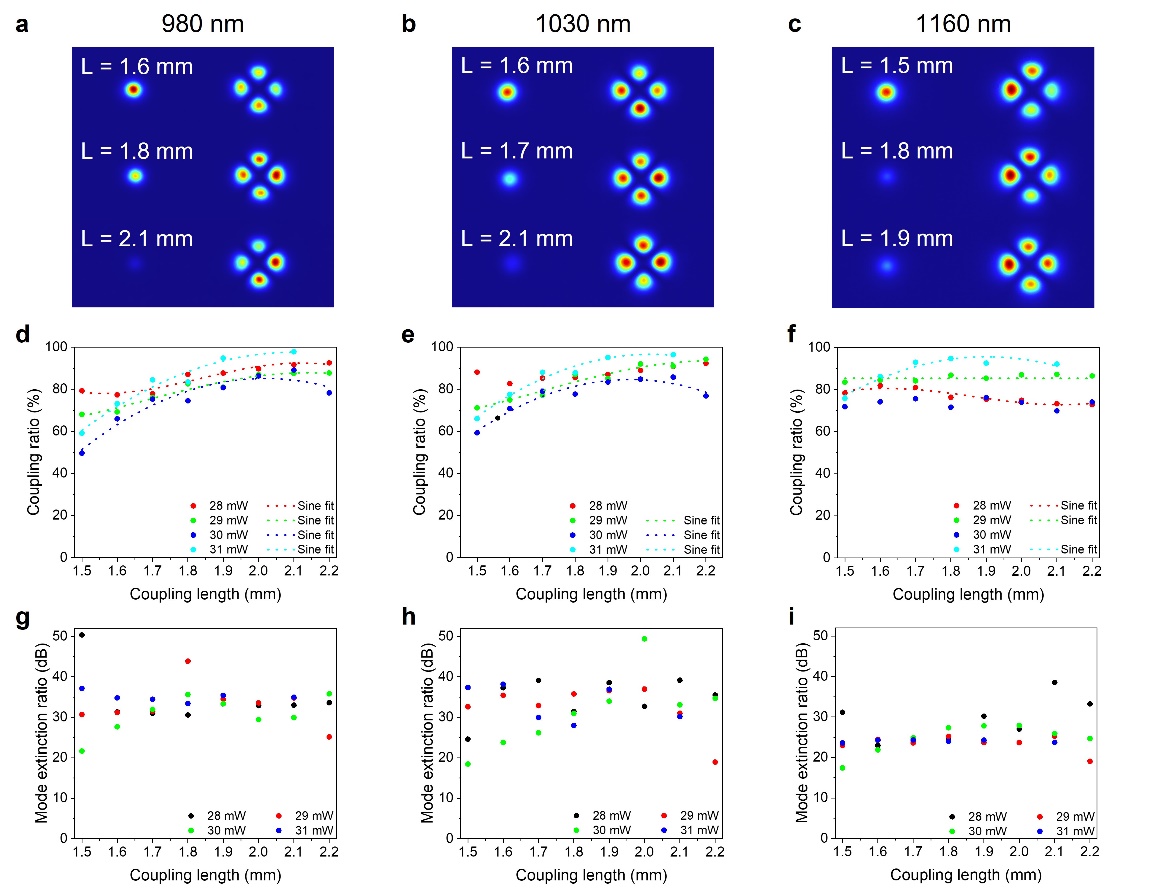


**Supplementary Fig. S17** LP_21_-mode directional coupler. **a-c**, Measured near-field distributions with various coupling length (L) at center wavelengths of 980 nm (a), 1030 nm (b) and 1160 nm (c). **d-f**, Measured coupling ratios with various coupling lengths (1.5-2.2 mm) at center wavelengths of 980 nm (d), 1030 nm (e) and 1160 nm (f). **g-i**, Measured mode extinction ratios of output LP_21_ modes at a coupling length of 1.5-2.2 mm at center wavelengths of 980 nm (g), 1030 nm (g) and 1160 nm (i).


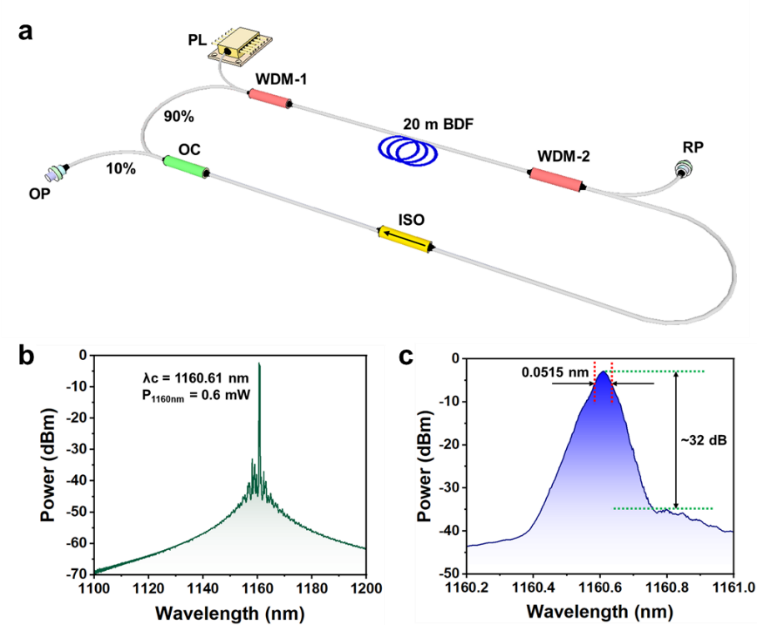


**Supplementary Fig. S18** 1160 nm laser. **a**, Setup for bismuth-doped fiber laser using a ring laser cavity. **b-c**, Optical spectrum (**b**) and spectral bandwidth (**c**) measurement for the laser.

**Supplementary text S12: Measured pulse autocorrelation after an objective and after a 30 mm length waveguide.**

A half-waveplate-polarizer combination is used for power control. The pulses are coupled with a microscope objective into the test waveguide to be measured. With a second microscope lens the fiber output can be directed into an optical spectrum analyzer (OSA) or an auto-correlator (**Fig. S19a-b**). The pulse duration is measured by the auto-correlator. The input pulse from the femtosecond laser has a duration of 225.3 fs (*Τ_p,in_*), which slightly extends to 225.8 fs (*Τ_p,out_*) after propagating through the glass waveguide with 30 mm length. Based on the pulse width measurements, we can estimate the nonlinear dispersion of the waveguide as follows^36^:

$T_{p,out}={[1+{({T_{c}}/{T_{p,in}})}^{4}]}^{1/2}\cdot T_{p,in}$, (4)

$T_{c}=2\cdot{(ln(2)\cdot\left| \varphi^{,,} \right|)}^{1/2}$, (5)

where, *Τ_c_* is the critical pulse width of ~58.4 fs, *φ^,^* is the group delay dispersion (GDD) of ~1230 fs^2^. Thus, the group velocity dispersion (GVD) is equal to 41 fs^2^ mm^-1^, and the dispersion parameter of the waveguide is -72.8 ps (nm km)^-1^ at a center wavelength of 1030 nm. **Fig. S19c** shows that the spectral width remains unchanged after the laser pulse propagates in the waveguide, except that the spectral distribution curve is superimposed with periodic fluctuations, which are considered to be caused by the interference of Fresnel reflected light from the front and rear end faces of the waveguide^37^. **Fig. S20** shows the measured MERs of the LP_21_ mode DC as a function of the spectral width of the input supercontinuum.


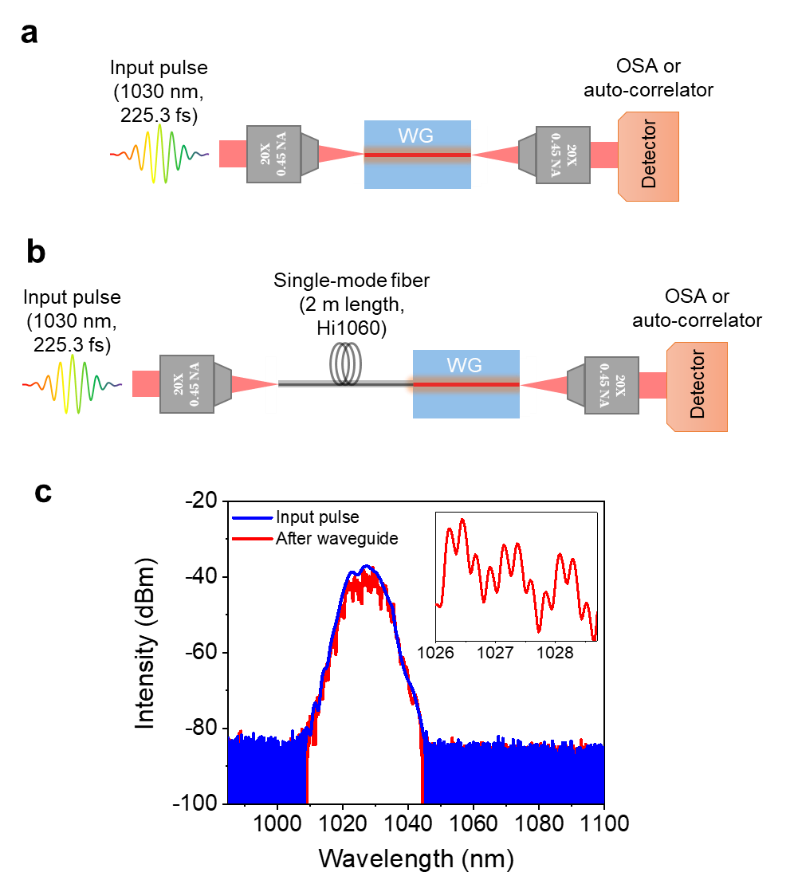


**Supplementary Fig. S19** Characterization of laser pulse before and after the waveguide. a-b, Schematic diagrams. c, Power spectrum measured before entering the waveguide and after propagating through the waveguide.


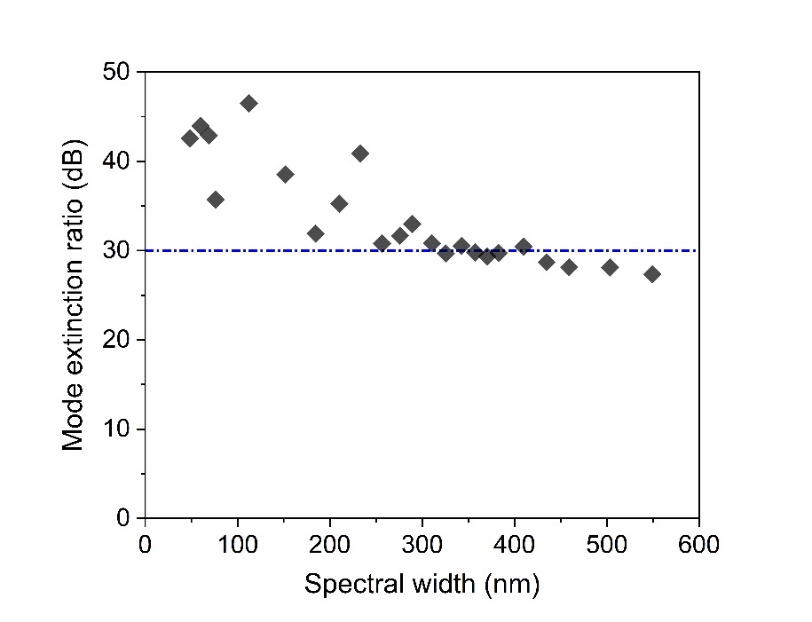


**Supplementary Fig. S20** Measured mode extinction ratios of LP_21_ mode DC as a function of the spectral width of the input supercontinuum.

**Supplementary text S13: Comparison of the OCMS-waveguide-based coupling structure with others.**

We compare the OCMS-waveguide-based coupling devices with conventional 2D planar waveguide coupling structures based on silicon-on-insulator (SOI)^38, 39, 40, 41, 42, 43, 44, 45, 46^, silicon nitride (Si_3_N_4_) ^47, 48, 49, 50, 51, 52^, and lithium niobate (LiNbO_3_)^53, 54, 55, 56^platforms. Table S1 summarizes key performances including coupling ratio variation, operating bandwidth and operating wavelength, as well as underlying mechanisms. It is obvious that the coupling devices based on glass 3D waveguides, including our work and the earlier work from the Withford group^25^, outperform even state-of-the-art 2D planar waveguide coupling devices, with an order of magnitude lower coupling ratio variation and operating wavelength covering visible and near-infrared spectrum. **Fig. S21** shows the overall advantages of our design, which simultaneously achieve broadband, high coupling ratio, as well as high robustness and low insertion loss (**Fig. 2**).

**Figure S21**. Comparison of OCMS-waveguide-based coupling structure with others.

**Supplementary Table S1. Comparison of OCMS-waveguide-based coupling structure based on glass 3D waveguides with others.**

| Platform | Bandwidth | Coupling ratio variation | Structural Design (Broadband Mechanism) | Ref. |
| --- | --- | --- | --- | --- |
| Glass 3D waveguide | 210 nm (931-1141 nm) | <0.1 dB | Directional coupler (Low dispersion substrate) | This work |
|  | 550 nm (817-1367 nm) | 0.5 dB | Directional coupler (Low dispersion substrate) | This work |
|  | 110 nm (1500-1610 nm) | <0.1 dB | Directional coupler (Low dispersion substrate) | This work |
|  | 450 nm (525-975 nm) | <0.1 dB | Tapered mode-selective coupler (Low dispersion substrate) | ^25^ |
| SOI 2D waveguide | 160 nm (1560-1620 nm) | 1 dB | Artificial gauge design (Periodic modulation) | ^38^ |
|  | 26-38 nm (Between 1520-1620 nm) |  | Straight waveguides | ^38^ |
|  | 47 nm (Between 1520-1620 nm) |  | Adiabatic design (Adiabatic mode evolution) | ^39, 40^ |
|  | 88 nm (Between 1520-1620 nm) |  | Asymmetric design (Phase match) | ^41^ |
|  | ~100 nm (Between 1520-1620 nm) |  |  | ^42^ |
|  | <71 nm (Between 1520-1620 nm) |  |  | ^43^ |
|  | 100 nm (Between 1520-1620 nm) | 1 dB | Metamaterials (Dispersion control by sub-wavelength structure) | ^44, 45^ |
|  | 120 nm (Between 1520-1620 nm) | 1 dB | Topological design (Topological protection) | ^46^ |
| Si_3_N_4_ 2D waveguide | 80 nm (centered at 1550 nm) | 1 dB | Directional coupler (Bending coupling) | ^48^ |
|  | 80 nm (1520-1600 nm) | 1 dB | Asymmetric directional couplers | ^49^ |
|  | 60 nm (around 1550 nm) | 3 dB | Grating coupling | ^50^ |
| Si_3_N_4_-on-Si 2D waveguide | 80 nm (1500-1580 nm) | 1 dB | Adiabatic design (Adiabatic mode evolution) | ^51^ |
|  | 35 nm (1530-1565 nm) | 1 dB | Asymmetric multimode interference | ^52^ |
| LiNbO_3_ 2D waveguide | 170 nm (around 1550 nm) | < 0.5 dB | Phase match by curved waveguides | ^54^ |
|  | 30 nm | 1 dB | Grating coupling | ^55^ |
|  | 50 nm (centered at 1555 nm) | 3 dB | Interferogram (electro-optic waveguide) | ^56^ |

## Supplementary Table 2. Corning Eagle XG glass refractive indices data

| Wavelength (nm) | Refractive index | Wavelength (nm) | **Refractive index** | Wavelength (nm) | Refractive index |
| --- | --- | --- | --- | --- | --- |
| 250 | 1.59021 | 770 | **1.50423** | 1290 | 1.49877 |
| 260 | 1.58176 | 780 | **1.50401** | 1300 | 1.49873 |
| 270 | 1.57445 | 790 | **1.50380** | 1310 | 1.49868 |
| 280 | 1.56808 | 800 | **1.50360** | 1320 | 1.49864 |
| 290 | 1.56247 | 810 | **1.50340** | 1330 | 1.49860 |
| 300 | 1.55752 | 820 | **1.50322** | 1340 | 1.49855 |
| 310 | 1.55312 | 830 | **1.50304** | 1350 | 1.49851 |
| 320 | 1.54919 | 840 | **1.50286** | 1360 | 1.49847 |
| 330 | 1.54566 | 850 | **1.50269** | 1370 | 1.49844 |
| 340 | 1.54248 | 860 | **1.50253** | 1380 | 1.49840 |
| 350 | 1.53960 | 870 | **1.50238** | 1390 | 1.49836 |
| 360 | 1.53699 | 880 | **1.50223** | 1400 | 1.49832 |
| 370 | 1.53461 | 890 | **1.50208** | 1410 | 1.49829 |
| 380 | 1.53244 | 900 | **1.50194** | 1420 | 1.49825 |
| 390 | 1.53045 | 910 | **1.50181** | 1430 | 1.49822 |
| 400 | 1.52862 | 920 | **1.50167** | 1440 | 1.49818 |
| 410 | 1.52693 | 930 | **1.50155** | 1450 | 1.49815 |
| 420 | 1.52537 | 940 | **1.50142** | 1460 | 1.49812 |
| 430 | 1.52393 | 950 | **1.50130** | 1470 | 1.49809 |
| 440 | 1.52259 | 960 | **1.50119** | 1480 | 1.49806 |
| 450 | 1.52135 | 970 | **1.50108** | 1490 | 1.49803 |
| 460 | 1.52020 | 980 | **1.50097** | 1500 | 1.49800 |
| 470 | 1.51912 | 990 | **1.50086** | 1510 | 1.49797 |
| 480 | 1.51811 | 1000 | **1.50076** | 1520 | 1.49794 |
| 490 | 1.51717 | 1010 | **1.50066** | 1530 | 1.49791 |
| 500 | 1.51629 | 1020 | **1.50057** | 1540 | 1.49788 |
| 510 | 1.51546 | 1030 | **1.50048** | 1550 | 1.49786 |
| 520 | 1.51468 | 1040 | **1.50039** | 1560 | 1.49783 |
| 530 | 1.51395 | 1050 | **1.50030** | 1570 | 1.49781 |
| 540 | 1.51326 | 1060 | **1.50021** | 1580 | 1.49778 |
| 550 | 1.51260 | 1070 | **1.50013** | 1590 | 1.49775 |
| 560 | 1.51199 | 1080 | **1.50005** | 1600 | 1.49773 |
| 570 | 1.51141 | 1090 | **1.49997** | 1610 | 1.49771 |
| 580 | 1.51086 | 1100 | **1.49990** | 1620 | 1.49768 |
| 590 | 1.51034 | 1110 | **1.49982** | 1630 | 1.49766 |
| 600 | 1.50984 | 1120 | **1.49975** | 1640 | 1.49764 |
| 610 | 1.50937 | 1130 | **1.49968** | 1650 | 1.49761 |
| 620 | 1.50892 | 1140 | **1.49961** | 1660 | 1.49759 |
| 630 | 1.50850 | 1150 | **1.49954** | 1670 | 1.49757 |
| 640 | 1.50810 | 1160 | **1.49948** | 1680 | 1.49755 |
| 650 | 1.50771 | 1170 | **1.49942** | 1690 | 1.49753 |
| 660 | 1.50734 | 1180 | **1.49936** | 1700 | 1.49751 |
| 670 | 1.50699 | 1190 | **1.49930** | 1710 | 1.49749 |
| 680 | 1.50666 | 1200 | **1.49924** | 1720 | 1.49747 |
| 690 | 1.50634 | 1210 | **1.49918** | 1730 | 1.49745 |
| 700 | 1.50604 | 1220 | **1.49913** | 1740 | 1.49743 |
| 710 | 1.50574 | 1230 | **1.49907** | 1750 | 1.49741 |
| 720 | 1.50546 | 1240 | **1.49902** | 1760 | 1.49740 |
| 730 | 1.50520 | 1250 | **1.49897** | 1770 | 1.49738 |
| 740 | 1.50494 | 1260 | **1.49892** | 1780 | 1.49736 |
| 750 | 1.50469 | 1270 | **1.49887** | 1790 | 1.49734 |
| 760 | 1.50446 | 1280 | **1.49882** | 1800 | 1.49733 |

**References:**

1. Eaton, S. M., et al. Heat accumulation effects in femtosecond laser-written waveguides with variable repetition rate. Optics Express **13**, 4708-4716 (2005).

2. Arriola, A.*, et al.* Low bend loss waveguides enable compact, efficient 3D photonic chips. *Optics Express* **21**, 2978-2986 (2013).

3. Schaffer, C. B., Jamison, A. O. & Mazur, E. Morphology of femtosecond laser-induced structural changes in bulk transparent materials. *Applied Physics Letters* **84**, 1441-1443 (2004).

4. Gattass, R. R. & Mazur, E. Femtosecond laser micromachining in transparent materials. *Nature Photonics* **2**, 219-225 (2008).

5. Sudrie, L.*, et al.* Femtosecond laser-induced damage and filamentary propagation in fused silica. *Physical Review Letters* **89**, 186601 (2002).

6. Lee, T.*, et al.* Low bend loss femtosecond laser written waveguides exploiting integrated microcrack. *Scientific Reports* **11**, 23770 (2021).

7. Chen, G. Y.*, et al.* Femtosecond-laser-written Microstructured Waveguides in BK7 Glass. *Scientific Reports* **8**, 10377 (2018).

8. Pan, Q.*, et al.* Nanocrystal-in-glass composite (NGC): A powerful pathway from nanocrystals to advanced optical materials. *Progress in Materials Science* **130**, 100998 (2022).

9. Chan, J. W.*, et al.* Modification of the fused silica glass network associated with waveguide fabrication using femtosecond laser pulses. *Applied Physics A* **76**, 367-372 (2003).

10. Terakado, N.*, et al.* A novel method for stress evaluation in chemically strengthened glass based on micro-Raman spectroscopy. *Communications Physics* **3**, 37 (2020).

11. Tan, D.*, et al.* Fabricating low loss waveguides over a large depth in glass by temperature gradient assisted femtosecond laser writing. *Optics Letters* **45**, 3941-3944 (2020).

12. Ponader, C. W., Schroeder, J. F. & Streltsov, A. M. Origin of the refractive-index increase in laser-written waveguides in glasses. *Journal of Applied Physics* **103**, 063516 (2008).

13. Poumellec, B.*, et al.* The UV-induced refractive index grating in Ge: preforms: additional CW experiments and the macroscopic origin of the change in index. *Journal of Physics D: Applied Physics* **29**, 1842 (1996).

14. Piao, F., Oldham, W. G. & Haller, E. E. Ultraviolet-induced densification of fused silica. *Journal of Applied Physics* **87**, 3287-3293 (2000).

15. Bressel, L.*, et al.* Femtosecond laser induced density changes in GeO2 and SiO2 glasses: fictive temperature effect [Invited]. *Optical Materials Express* **1**, 605-613 (2011).

16. Atkins, P., Atkins, P. W. & de Paula, J. *Atkins' physical chemistry*. (New York, Oxford university press, 2014).

17. Lijing, Z.*, et al.* Porous glass density tailoring by femtosecond laser pulses. *Optical and Quantum Electronics* **52**, 1-8 (2020).

18. Kostyuk, G. K., Sergeev, M. M. & Yakovlev, E. B. The processes of modified microareas formation in the bulk of porous glasses by laser radiation. *Laser Physics* **25**, 066003 (2015).

19. Szameit, A.*, et al.* Two-dimensional soliton in cubic fs laser written waveguide arrays in fused silica. *Optics Express* **14**, 6055-6062 (2006).

20. Mansour, I. & Caccavale, F. An improved procedure to calculate the refractive index profile from the measured near-field intensity. *Journal of Lightwave Technology* **14**, 423-428 (1996).

21. Ngcobo, S.*, et al.* A digital laser for on-demand laser modes. *Nature Communications* **4**, 2289 (2013).

22. Hodgson, N. & Weber, H. Laser Resonators and Beam Propagation: Fundamentals, Advanced Concepts, Applications. Springer (2005).

23. Wan, Z.*, et al.* Divergence-degenerate spatial multiplexing towards future ultrahigh capacity, low error-rate optical communications. *Light: Science & Applications* **11**, 144 (2022).

24. Love, J. D. & Riesen, N. Mode-selective couplers for few-mode optical fiber networks. *Optics Letters* **37**, 3990-3992 (2012).

25. Gross, S.*, et al.* Three-dimensional ultra-broadband integrated tapered mode multiplexers. *Laser & Photonics Reviews* **8**, L81-L85 (2014).

26. Lau, K. Y., Liu, X. & Qiu, J. MXene Saturable Absorbers in Mode-Locked Fiber Laser. *Laser & Photonics Reviews* **16**, 2100709 (2022).

27. Zhang, L.*, et al.* ‘Plug-and-play’ plasmonic metafibers for ultrafast fibre lasers. *Light: Advanced Manufacturing* **3**, 45 (2022).

28. Snyder, A. W. & Love, J. *Optical waveguide theory*. Springer Science & Business Media (2012).

29. Huang, Q. & Chiang, K. S. High-Order-Mode-Pass Mode (De)Multiplexer With a Hybrid-Core Vertical Directional Coupler. *Journal of Lightwave Technology* **37**, 3932-3938 (2019).

30. Haus, H. A. & Huang, W. Coupled-mode theory. *Proceedings of the IEEE* **79**, 1505-1518 (1991).

31. Huang, W.-P. Coupled-mode theory for optical waveguides: an overview. *Journal of the Optical Society of America A* **11**, 963-983 (1994).

32. Haus, H.*, et al.* Coupled-mode theory of optical waveguides. *Journal of Lightwave Technology* **5**, 16-23 (1987).

33. Hardy, A. & Streifer, W. Coupled mode theory of parallel waveguides. *Journal of Lightwave Technology* **3**, 1135-1146 (1985).

34. Thornburg, W. Q., Corrado, B. J. & Zhu, X. D. Selective launching of higher-order modes into an optical fiber with an optical phase shifter. *Optics Letters* **19**, 454-456 (1994).

35. Riesen, N. & Love, J. D. Weakly-Guiding Mode-Selective Fiber Couplers. *IEEE Journal of Quantum Electronics* **48**, 941-945 (2012).

36. Weiner, A. M. *Ultrafast optics*. John Wiley & Sons (2011).

37. Ferreira, P. H. D.*, et al.* Improvement in measuring losses by interferometric technique for glass waveguides produced by femtosecond laser writing. *Optics Communications*, 129132 (2022).

38. Song, W.*, et al.* Dispersionless Coupling among Optical Waveguides by Artificial Gauge Field. *Physical Review Letters* **129**, 053901 (2022).

39. Yun, H., et al. 2x2 adiabatic 3-dB coupler on silicon-on-insulator rib waveguides. SPIE (2013).

40. Mrejen, M.*, et al.* Adiabatic elimination-based coupling control in densely packed subwavelength waveguides. *Nature Communications* **6**, 7565 (2015).

41. Lu, Z.*, et al.* Broadband silicon photonic directional coupler using asymmetric-waveguide based phase control. *Optics Express* **23**, 3795-3808 (2015).

42. Chen, S.*, et al.* Low-loss and broadband 2×2 silicon thermo-optic Mach–Zehnder switch with bent directional couplers. *Optics Letters* **41**, 836-839 (2016).

43. Chen, G. F. R.*, et al.* Broadband Silicon-On-Insulator directional couplers using a combination of straight and curved waveguide sections. *Scientific Reports* **7**, 7246 (2017).

44. Halir, R.*, et al.* Colorless directional coupler with dispersion engineered sub-wavelength structure. *Optics Express* **20**, 13470-13477 (2012).

45. Wang, Y.*, et al.* Compact Broadband Directional Couplers Using Subwavelength Gratings. *IEEE Photonics Journal* **8**, 1-8 (2016).

46. Song, W.*, et al.* Robust and Broadband Optical Coupling by Topological Waveguide Arrays. *Laser & Photonics Reviews* **14**, 1900193 (2020).

47. Sharma, T.*, et al.* Review of Recent Progress on Silicon Nitride-Based Photonic Integrated Circuits. *IEEE Access* **8**, 195436-195446 (2020).

48. Li, D.*, et al.* Broadband Silicon Nitride Power Splitter Based on Bent Directional Couplers with Low Thermal Sensitivity. *Micromachines* **13**, 559 (2022).

49. Bhandari, B.*, et al.* Highly efficient broadband silicon nitride polarization beam splitter incorporating serially cascaded asymmetric directional couplers. *Optics Letters* **45**, 5974-5977 (2020).

50. Chen, Y.*, et al.* Experimental demonstration of an apodized-imaging chip-fiber grating coupler for Si3N4 waveguides. *Optics Letters* **42**, 3566-3569 (2017).

51. Sacher, W. D.*, et al.* Polarization rotator-splitters and controllers in a Si3N4-on-SOI integrated photonics platform. *Optics Express* **22**, 11167-11174 (2014).

52. Sun, X., Aitchison, J. S. & Mojahedi, M. Realization of an ultra-compact polarization beam splitter using asymmetric MMI based on silicon nitride / silicon-on-insulator platform. *Optics Express* **25**, 8296-8305 (2017).

53. Chen, G.*, et al.* Advances in lithium niobate photonics: development status and perspectives. *Advanced Photonics* **4**, 034003 (2022).

54. Zhang, L.*, et al.* Compact, Broadband and Low-Loss Polarization Beam Splitter on Lithium-Niobate-On-Insulator Using a Silicon Nanowire Assisted Waveguide. *IEEE Photonics Journal* **12**, 1-6 (2020).

55. Chen, B.*, et al.* Two-dimensional grating coupler on an X-cut lithium niobate thin-film. *Optics Express* **29**, 1289-1295 (2021).

56. Pohl, D.*, et al.* An integrated broadband spectrometer on thin-film lithium niobate. *Nature Photonics* **14**, 24-29 (2020).
